# Deciphering antifungal and antibiofilm mechanisms of isobavachalcone against *Cryptococcus neoformans* through RNA-seq and functional analyses

**DOI:** 10.1186/s12934-024-02369-2

**Published:** 2024-04-12

**Authors:** Weidong Qian, Jiaxing Lu, Chang Gao, Qiming Liu, Yongdong Li, Qiao Zeng, Jian Zhang, Ting Wang, Si Chen

**Affiliations:** 1 School of Biological and Pharmaceutical Engineering, Shaanxi University of Science and Technology, Xi’an, 710021 P. R. China; 2 Ningbo Municipal Center for Disease Control and Prevention, Ningbo, 315010 P. R. China; 3 School of Pharmaceutical Sciences, Shenzhen University Medical School, Shenzhen, 518060 China; 4 Department of Immunology, Shenzhen University Medical School, Shenzhen, 518060 China

**Keywords:** *Cryptococcus neoformans*, Isobavachalcone, Antifungal activity, RNA-sequencing, Functional profiling

## Abstract

*Cryptococcus neoformans* has been designated as critical fungal pathogens by the World Health Organization, mainly due to limited treatment options and the prevalence of antifungal resistance. Consequently, the utilization of novel antifungal agents is crucial for the effective treatment of *C. neoformans* infections. This study exposed that the minimum inhibitory concentration (MIC) of isobavachalcone (IBC) against *C. neoformans* H99 was 8 µg/mL, and IBC dispersed 48-h mature biofilms by affecting cell viability at 16 µg/mL. The antifungal efficacy of IBC was further validated through microscopic observations using specific dyes and in vitro assays, which confirmed the disruption of cell wall/membrane integrity. RNA-Seq analysis was employed to decipher the effect of IBC on the *C. neoformans* H99 transcriptomic profiles. Real-time quantitative reverse transcription PCR (RT-qPCR) analysis was performed to validate the transcriptomic data and identify the differentially expressed genes. The results showed that IBC exhibited various mechanisms to impede the growth, biofilm formation, and virulence of *C. neoformans* H99 by modulating multiple dysregulated pathways related to cell wall/membrane, drug resistance, apoptosis, and mitochondrial homeostasis. The transcriptomic findings were corroborated by the antioxidant analyses, antifungal drug sensitivity, molecular docking, capsule, and melanin assays. In vivo antifungal activity analysis demonstrated that IBC extended the lifespan of *C. neoformans*-infected *Caenorhabditis elegans*. Overall, the current study unveiled that IBC targeted multiple pathways simultaneously to inhibit growth significantly, biofilm formation, and virulence, as well as to disperse mature biofilms of *C. neoformans* H99 and induce cell death.

**Supplementary Information:**

The online version contains supplementary material available at 10.1186/s12934-024-02369-2.

## Introduction

Fungal pathogens pose an escalating risk to global health, inflicting significant disease on over a billion individuals worldwide [1]. Invasive fungal disease (IFD) is responsible for causing more fatalities annually than either tuberculosis or malaria. This seminal review highlights the profound impact that fungal pathogens have on human health. Nevertheless, the influence of fungal infections on human health and beyond is an ever-increasing issue, although often underestimated [2]. Moreover, the number of antifungal agents available for treating human fungal infections, including polyenes, echinocandins, flucytosine, and azoles, is poorly limited [3]. Polyenes disrupt the plasma membrane by binding to ergosterol, an essential component of the fungal cell membrane [4]. In contrast, azoles target and block ergosterol biosynthetic pathway by suppressing the function of lanosterol 14α-demethylase, a key enzyme in fungal ergosterol biosynthesis [5]. Echinocandins, the only licensed antifungal agents that target the cell wall, compromise cell wall integrity by inhibiting the production of the (1,3)-β-D-glucan, a fundamental structural component of the fungal cell wall [6]. The situation is further complicated by the rapid emergence of clinical pathogens resistance to antifungals, due to the extensive use of existing antifungals in agricultural and clinical settings. Recent evidence indicates that eco-environmental changes and increased international travel and trade have altered or expanded the incidence and geographic distribution of fungal diseases globally [7].

In response to the escalating challenge of IFD and increased antifungal resistance, the World Health Organization (WHO) introduced the Fungal Priority Pathogens List (FPPL) on October 25, 2022. This initiative aims to strengthen the global response to fungal infections and antifungal resistance. The WHO FPPL identifies 19 fungal priority pathogens that pose the most significant threat to public health. Among these, *Cryptococcus neoformans*, *Candida auris*, *Aspergillus fumigatus*, and *Candida albicans* were ranked and categorized as critical fungal pathogens based on certain factors such as annual incidence, trends in incidence and prevalence, global distribution, risk of complications, antifungal resistance, and access to testing and evidence-based treatment options. *C. neoformans*, the top-ranked fungal pathogen WHO FPPL, is of particular concern due to its global distribution and ability to cause life-threatening cryptococcosis [7]. Like other opportunistic fungi, immunocompromised individuals are most vulnerable to *C. neoformans* infections [8]. The incidence of cryptococcal infections remains high in these individuals, with cryptococcal meningitis being the leading cause of death among HIV-infected individuals, second only to tuberculosis [9]. For example, an estimated 223,100 cases of cryptococcal meningitis were reported worldwide in 2014, resulting in approximately 181,100 deaths [10]. Another critical issue is the emergence of antifungal-resistant clinical isolates; the antifungal resistance mechanisms remain largely unexplored.

*C. neoformans* is predominantly isolated from avian excreta, especially pigeons [11]. As a soil-dwelling organism, *C. neoformans* typically exists in communities of clustered microbial cells surrounded by an environmental matrix. Indeed, the microbial communities, known as biofilms, represent the predominant form for up to 80% of microbial cells in the natural environment [12]. Polysaccharides are the major and the most essential component of the biofilm matrix [13]. Under certain conditions, *C. neoformans* produces a thick capsule layer composed of extracellular polysaccharides, with glucuronoxylomannan (GXM) being the main component of the polysaccharide capsule [14]. Prior research has indicated that GXM plays a pivotal role in forming *C. neoformans* biofilm, and its production is closely associated with pathogenicity and virulence [15]. Like most microbial biofilms, biofilm-associated cryptococcal cells exhibit more excellent resistance to immune elimination, clearance, and antifungal therapy than their planktonic counterparts [16]. Furthermore, *C. neoformans* frequently forms structured biofilms on the surface of medical device, such as the prosthetic heart valve, the ventriculo-arterial shunt catheters, and the prosthetic joint [17], making the treatment of medical devices-related infections particularly challenging, and leading to frequent relapses of cryptococcal infections. Therefore, the discovery of novel, safe, and effective antifungal agents to combat infections caused by the planktonic cells and biofilms of *C. neoformans* H99 is of utmost importance.

Conventionally, natural substances derived from plants used in traditional medicine to treat infectious diseases have been a primary source of innovative therapeutic lead compounds [18]. Consequently, many natural products have been thoroughly investigated for their ability to inhibit or eliminate a broad spectrum of clinically significant human pathogens [19]. One such natural compound, isobavachalcone (IBC), is primarily extracted from the seeds of *Psoralea corylifolia* Linn., a plant commonly used in traditional medicines in China and India [20]. IBC has been found to exhibit various pharmacological properties, including anticancer [21], antibacterial [20], anti-inflammatory [22], antiviral [20], neuroprotective [23], and bone-protective effects [24]. However, the antifungal and antibiofilm efficacy of IBC against *C. neoformans* H99, and the underlying mechanisms of action remain unexplored. Against this backdrop, the present study was designed to examine the antifungal and antibiofilm potential of IBC against *C. neoformans* H99. Furthermore, the study aimed to decipher the molecular mechanism underlying the antifungal and antibiofilm efficacy of IBC. This research could contribute for developing novel therapeutic strategies in treating infections caused by *C. neoformans*, particularly in the context of increasing antifungal resistance.

## Materials and methods

### Reagents

IBC, with a purity of 98% or higher, was obtained from Chengdu Lemeitian Pharmaceutical Technology Co. Ltd, China. It was dissolved in 5% dimethyl sulfoxide (DMSO) to prepare a 10 mg/mL stock solution, and stored at 4 °C for further use. Other chemicals, including rotenone, SHAM, carboxin, antimycin A and NaN_3_ were procured from Sigma-Aldrich (Shanghai, China). Rotenone and SHAM were dissolved in DMSO, carboxin was reconstituted in acetone, antimycin A was reconstituted in 95% ethanol, and NaN_3_ was reconstituted in water. Dyes such as SYTO 9, FUN®1, Calcofluor® White M2R stain (CWS), and propidium iodide (PI) were purchased from Invitrogen, a part of Thermo Fisher Scientific, Massachusetts, USA.

### Fungal strain and growth conditions

*C. neoformans* H99 strain was cultivated in yeast extract-peptone-2% dextrose (YPD) (Sigma-Aldrich, USA), and incubated at 30 °C. The strain was preserved at − 80 °C in 20% glycerol solution, and frozen stocks were recovered on YPD agar medium at 30 °C for a duration of 36 h. A single fresh single colony of *C. neoformans* H99 was incubated into YPD, cultured overnight at 30 °C with a shaking at 150 rpm, and diluted to produce a solution with a concentration of either 10^3^ or 10^6^ cells/mL as required for the experiments.

### Determination of the minimum inhibitory concentration (MIC) of IBC

The broth microdilution method was used to determine the MIC of IBC against *C. neoformans* H99. IBC was introduced in a 2-fold serial dilution, starting at a concentration of 256 µg/mL, into each well of the 96-well plate. Each well contained 200 µL of YPD and a final dilution of 10^4^ cells/mL from an overnight culture of *C. neoformans* H99. The plate was incubated at 30 °C for a period of 24 h. Following incubation, the turbidity of the culture was measured at 600 nm (OD_600_). The MIC was identified as the lowest concentration of IBC that resulted in maximum growth inhibition.

### Growth curve analysis

To assess the inhibitory effect of IBC on the growth of *C. neoformans* H99, IBC at a concentration of 8 µg/mL was added into 3 ml of YPD broth containing 2% of a freshly cultured overnight sample of *C. neoformans* H99. For comparison, a negative control was established by inoculating 3 ml of YPD broth with 1% of a fresh overnight culture of *C. neoformans* H99, without the addition of IBC. Subsequently, the tubes were incubated at 30 °C for 24 h. The turbidity at OD_600_ of both treated and untreated samples was measured at regular intervals of 2 h [25].

### Changes in cell wall/membrane, metabolic activity, morphology, plasma membrane potential, and ergosterol levels

Changes in cell wall/membrane, viability and morphology in *C. neoformans* H99 cells treated with and without IBC were analyzed both qualitatively and quantitatively using various techniques. The samples were prepared in a tube containing 2 mL of YPD with an initial cell concentration of 1 × 10^6^ cells/mL, and subjected to various concentrations (0 MIC, 1 MIC and 2 MIC) of IBC. Next, the assay plate was incubated at 30 °C for either 6 or 24 h, depending on the specific requirements of the experiment. After incubation, the samples were evaluated using a range of techniques, including microscopes, and biochemical analysis. All experiments were performed in triplicate to ensure the reliability of the results.

#### Confocal laser scanning microscopy (CLSM) analysis

The changes in cell membrane were evaluated using CLSM (Zeiss LSM-880, Oberkochen, Germany), the IBC-treated and untreated cells were cultivated for 24 h, washed with sterile phosphate-buffered saline (PBS), and resuspended in PBS. The resulting cultures were harvested by centrifugation at 6000 rpm for 15 min, gently washed twice with PBS, and resuspended in PBS. A combination of 2.5 µM SYTO 9 and 5 µM PI was mixed with 0.1 mL of treated or untreated cell suspensions, and the samples were incubated in the dark at 25 °C for 20 min. After incubation, the samples were imaged using a CLSM, with fluorescence excitation/emission wavelengths of 488/520 nm for SYTO 9 and 535/617 nm for PI, respectively. Changes in cell membrane permeability were proportional to the reduction in the level of green color to red [26].

The changes in metabolic activity were examined using CLSM, a mixture of 20 mM FUN®1 and 3 mM CWS was mixed with 0.1 mL of treated or untreated cell suspensions, and the samples were incubated in the dark at 30 °C for 45 min. The samples were imaged using a CLSM, with fluorescence excitation/emission wavelengths of 470/590 nm for FUN®1 and 488/617 nm for CWS, respectively [27].

#### Field emission scanning electron microscopy (FESEM) and transmission electron microscope (TEM) analysis

The changes in cell wall and morphology were evaluated using FESEM (Nova Nano SEM-450, FEI, Hillsboro, USA) analysis, the IBC-treated and untreated cells were washed with sterile PBS after 6 h of treatment. Then, these cells were pretreated with 2.5% glutaraldehyde at 4 °C for 4 h, and washed once with sterile PBS. The prefixed cells were sequentially dehydrated with increasing concentrations of ethanol (30, 50, 70, 80, 90, and 100%), further subjected to isoamyl acetate at 25 °C for 1 h and air dried. Finally, the treated cells were sputter-coated using an ion sputter, and evaluated using a FESEM [26].

For TEM (HITACHI HT7800) analysis, the IBC-treated and untreated cells were washed three times with sterile PBS after 6 h of treatment, and then pretreated with 2.5% glutaraldehyde at 4 °C. After a 12-h incubation, the cells were collected and washed twice with PBS, and fixed with 1% osmium acid solution for 2 h at 4 °C. The cells were dehydrated in increasing gradients of ethanol concentration (50%, 70%, 80%, 85%, 90%, 95% and 100%), and treated with acetone for 20 min. The sample was further treated with a 1:3 mixture of acetone and epoxy resin, and subsequently embedded in ethoxylated resin. The resulting samples were cut into ultra-thin Sects. (70–90 nm thick) using a diamond slicer, and stained for 5–10 min each with lead citrate solution and 50% ethanol-saturated solution of UO_2_ acetate, respectively. After air-drying, the TEM was used to observe the changes in cell wall/morphology [28].

#### Microliter multimode plate reader analysis

To examine changes in plasma membrane potential, fungal cells were cultured, and treated with various concentrations of IBC (0, 1/4, 1/2, 1, 3/2 and 2 MIC) at 30 °C for 4 h. Subsequently, fungal cells were collected by centrifugation at 4000 × g for 15 min, washed twice with PBS, and resuspended in 200 µL of Bis-(1,3-Dibutylbarbituric Acid Trimethine Oxonol) (DiBAC_4_(3), Molecular Probes, Eugene, OR, USA) in PBS. After incubation for 20-minute incubation at 25 °C, the fluorescence values were determined using a microliter multimode plate reader (Perkin Elmer, USA), with excitation/emission wavelengths of 490 and 516 nm, respectively [29].

#### Ergosterol extraction and quantification using a spectrophotometer

Ergosterol quantification was conducted to compare the total ergosterol yield in samples from both the untreated and treated group samples. The total sterols in IBC-treated and untreated *C. neoformans* H99 cells were extracted using a method described in previous research [30]. Initially, a fresh overnight YPD broth culture of *C. neoformans* H99 was used to inoculate 3 mL of YPD broth to achieve a concentration of approximately 10^6^ cells/mL, and subjected to various concentrations of IBC for an incubation of 18 h. The cultures were collected by centrifugation at 5000× g for 15 min, and the net weight of the cell pellets was determined. 3 mL of 25% ethanolic potassium hydroxide solution were added to each pellet, vortex-mixed for 5 min, and incubated at 85 °C for 1 h. Then, a mixture of 1 mL of sterile water and 3 mL of n-heptane was added to the cell suspension to extract intracellular sterols. After a vigorous vortex-mixing for 5 min, the n-heptane layer was collected and the absorbance between 230 and 300 nm was measured in a spectrophotometer. The ergosterol content was calculated as a percentage of the wet weight of the cell.

### Microscopic analyses of *C. Neoformans* H99 biofilms

The biofilm structure of samples treated with and without IBC was qualitatively analyzed using various microscopic techniques. The biofilm assay was conducted on a glass slide placed in a 24-well plate containing 1 mL of YPD, with an initial cell concentration of 1 × 10^8^ cells/mL. To examine the anti-adhesion effect of IBC, the cells were subjected to various concentrations (0 MIC, 1/4 MIC, 1/2 MIC, and 1 MIC) of IBC, and the assay plate was incubated at 30 °C for 4 h. Conversely, for the biofilm inhibition analysis, the assay plate was incubated at 30 °C for 24 h. Furthermore, to assess the dispersal effect of IBC on mature biofilms, the assay plate was initially incubated at 30 °C for 48 h to allow the formation of mature biofilms in the absence of IBC. The mature biofilms were subjected to various concentrations (0 MIC, 2 MIC, 4 MIC, and 8 MIC) of IBC at 30 °C for 6 h. After incubation, the resulting biofilms were stained, and imaged using microscopes.

#### Light microscopic examination

For examination under light microscopic, the biofilms formed on the surface of glass slide were gently washed twice with sterile PBS, and stained with 0.1% (w/v) crystal violet (CV) for 20 min. The biofilms were rinsed once with sterile PBS to remove excess stains. The evaluation of biofilms was performed using a light microscope at a magnification of 400×. For biofilm quantitative examination, the biofilm biomass was determined using the CV assay as described previously [26].

#### CLSM analysis

For CLSM analysis, the biofilms formed on the surface of the glass slide were washed with sterile PBS, and stained with a mixture of 2.5 µM SYTO 9 and 5 µM PI under dark conditions for 15 min. The resulting samples were rinsed once with sterile PBS to remove unbound stains, and air dried. The evaluation of biofilms was conducted using a CLSM at a magnification of 200×.

#### FESEM analysis

For FESEM analysis, the biofilms formed on the surface of glass slide were washed with sterile PBS, prefixed with 2.5% glutaraldehyde at 4 °C for 4 h, and washed once with sterile PBS. The prefixed biofilms were sequentially treated as described above. Then the treated biofilms were sputter-coated using an ion sputter, and examined using a FESEM.

### IBC effects on mitochondrial morphology, mitochondrial membrane potential (MMP), mitochondrial ATP, mitochondrial reactive oxygen species (ROS), and the electron transport chain

The effects of IBC on mitochondrial morphology, MMP, mitochondrial ATP, ROS and the electron transport chain were evaluated both qualitatively and quantitatively using multiple techniques. Samples were prepared in each well of a 6-well plate containing 3 mL of YPD and an initial cell concentration of 1 × 10^6^ cells/mL, and were treated with different concentrations of IBC. The assay plate was incubated at 30 °C for either 12 or 24 h, depending on the specific requirements of the experiment. After incubation, the cells were collected, and evaluated using various techniques, including biochemical analysis, the spot plate assay and microscopes. All experiments were performed in triplicate to ensure the reliability of the results.

#### CLSM for mitochondrial morphology analysis

The *C. neoformans* H99 strain was cultured in YPD broth medium at 30 °C for 18 h in the presence of various concentrations of IBC (0 MIC, 1/4 MIC, 1/2 MIC and 1 MIC). Approximately 10^6^ cells/mL from each sample were stained with 250 nM Mito-Tracker Red CMXRos (Beyotime) for 30 min at 30° C with shaking at 150 rpm. Next, the cells were harvested, washed twice with PBS and imaged using CLSM. The characterization of mitochondrial morphology was performed in accordance with a previous report [31].

#### MMP analysis

The *C. neoformans* H99 strain was cultured in YPD broth medium at 30 °C for 18 h in the presence of various concentrations of IBC (0 MIC, 1/4 MIC, 1/2 MIC, 1 MIC, 3/2 MIC and 2 MIC). The cells were collected and pelleted. The changes in MMP were determined using a MMP assay kit with JC-10 (Ywasen, China) according to the manufacturer’s instructions. The average values of fluorescence intensities of the JC-10 monomers (green fluorescence emission at 525 nm) were determined by measuring the emission wavelength of 525 nm using a microliter Multimode Plate Reader [32].

#### Mitochondrial ATP quantification analysis

The *C. neoformans* H99 strain was cultured in YPD broth medium at 30 °C for 18 h in the presence of various concentrations of IBC (0 MIC, 1/4 MIC, 1/2 MIC, 1 MIC, 3/2 MIC and 2 MIC). The cells were collected, frozen on dry ice, and lysed by bead beating with 0.5 mm zirconium beads, as previously described [33]. The resulting lysates were mixed with 1.4 mL NP40 lysis buffer (6 mM Na_2_HPO_4_, 4 mM NaH_2_PO_4_, 1% Nonidet P-40, 150 mM NaCl, 2 mM EDTA, 1× protease inhibitors, 1× phosphatase inhibitors, and 1 mM phenylmethylsulfonyl fluoride). The mixtures were centrifuged at 2500× g for 5 min at 4 °C to remove smaller cellular debris. A firefly luciferase bioluminescent assay (Invitrogen) was used to quantify total intracellular ATP according to standard protocol. Moreover, total protein concentrations of cell lysates were determined and normalized using a bicinchoninic acid (BCA) assay. Statistical significance in total intracellular ATP were determined with a two-way analysis of variance (ANOVA).

#### ROS analysis

The ROS in IBC-treated and -untreated fungal cells, 2′,7′-dichlorodihydrofluorescein diacetate (DCFH-DA) (Beyotime, China) was assessed using a fluorescent redox probe. The fungal cells were washed twice with PBS, and mixed with dihydroethidium in PBS for 30–40 min at 30 °C. Next, the treated cells were harvested, and resuspended in 1 mL of pre-warmed PBS. Finally, the fluorescence values were measured using a microliter multimode plate reader, with excitation/emission wavelengths of 488/525 nm [34].

#### Spot plate assay for IBC effect determination on electron transport chain

The electron transport chain in mitochondria is a regulated process performed by four protein complexes that mediate redox reactions, culminating in the production of ATP through the process of oxidative phosphorylation. To assess the impact of IBC on this process, the *C. neoformans* H99 strain was incubated in YPD broth medium at 30 °C for 18 h, either in the presence or absence of sub-MIC IBC. The cells were collected, rinsed once with PBS, and resuspended in PBS. The suspensions were 10-fold-diluted, and 5 µL of serial dilutions were spotted onto YPD agar medium supplemented with various inhibitors of the electron transport chain. These included the rotenone (an inhibitor of Complex I) at 0.5 mg/mL, carboxin (an inhibitor of Complex II) at 50 µg/mL, antimycin (an inhibitor of Complex III) at 3 µg/mL and sodium azide (an inhibitor of Complex IV) at 0.5 mM, as well as salicylhydroxamic acid (SHAM, an inhibitor of alternative oxidase) at 2.5 mM. The plates were incubated at 30 °C for 48 h and imaged [35].

### Annexin -FITC/PI double staining of apoptosis

Overnight cultures of *C. neoformans* H99 (∽ 10^8^ cells/mL) were rinsed within PBS and used to inoculate 3 mL cultures of RPMI 1640 medium with 165 mM MOPS (pH 7) and various final concentrations of IBC. The cultures were incubated at 30 °C with shaking at 160 rpm for 12 h. The cells were harvested, washed once with 1 mL of Annexin V binding buffer (10 mM HEPES-NaOH, pH 7.4, 140 mM NaCl, and 2.5 mM CaCl_2_), and resuspended in 25 µL of Annexin V binding buffer. A mixture of 5 µL of FITC-conjugated annexin V and PI was then added and incubated at 25 °C. After a 15-minute incubation, the cells were collected and resuspended in PBS. The resuspensions were placed onto a microscope slide with a coverslip, and imaged using CLSM. Representative fields were taken, and the images were obtained using 488-nm excitation for FITC-conjugated annexin V [36].

### Synergistic activity of IBC and fluconazole

The synergistic activity was assessed using the microtiter plate method. Stock solutions of IBC and fluconazole were serially diluted in RPMI 1640 in various combinations to achieve final concentrations ranging from 0 MIC to 1/2 MIC for IBC and from 0.25 to 1 µg/mL for fluconazole. A fresh overnight RPMI 1640 broth culture of *C. neoformans* H99 was added to each well of a 24-well plate at a final concentration of approximately 2.1 × 10^3^ cells/mL and incubated for 24 h at 30 °C. This was followed by ten-fold serial dilutions, with 5 µL from each dilution spotted on YPD agar plates and incubated at 30 °C on YPD plates alone or containing multiple concentrations of IBC as indicated. Images were captured at 48 h. All assays were conducted in triplicate. Moreover, the fractional inhibitory concentration index (FICI) was employed to examine the synergistic activity between IBC and fluconazole, and was calculated using formula described in a previous report [37].

### Capsule production

Overnight cultures of *C. neoformans* H99 (∽ 10^8^ cells/mL) were collected by centrifugation, rinsed twice with sterile PBS, followed by resuspending in PBS. The cell suspensions were diluted to a concentration of 10^6^ cells/mL in RPMI 1640 with 165 mM MOPS (pH 7) and incubated for 24 h at 30 °C in the presence of various concentrations of IBC. Then, 10 µL aliquots of the cell suspension were mixed with one drop of India Ink, and the capsule was visualized using microscopy. The images were captured for at least 50 cells for each of the different concentrations, and the statistical analysis for the capsule sizes among samples was conducted [38].

### Melanization assay

Overnight cultures of *C. neoformans* H99 (∽ 10^8^ cells/mL) were cultured at specified concentrations of IBC at 30 °C for 24 h. The cells were collected by centrifugation, rinsed twice with sterile PBS, and resuspended in PBS. A 20 µL aliquot from the cell suspensions was spotted onto solid L-DOPA media (7.6 mM L-asparagine monohydrate, 5.6 mM glucose, 22 mM KH_2_PO_4_, 1 mM MgSO_4_•7H_2_0, 0.5 mM L-DOPA, 0.3 mM thiamine-HCl, 20 nM biotin, 2% agar) containing various concentrations of IBC, incubated at 30 °C, for 3–7 d. Visual analysis of melanin production was conducted and photographed. Moreover, the relative quantitative analysis of melanin production was performed using image J software according to a previous report [39].

### In vitro phenotypic assays

The stress assays on *C. neoformans* H99 were conducted in vitro, following the method as described in a previous study [40]. The organism was initially cultivated in yeast extract peptide dextrose (YPD) medium and incubated overnight at 30 °C. Then, the cells were collected, rinsed three times with PBS, and diluted to a concentration of 1 × 10^8^ cells/mL in PBS. Subsequently, 5 µL aliquots of serial dilutions were spotted on YPD agar medium supplemented with numerous stress-inducing compounds. These compounds included Calcofluor white (1.5 mg/mL), caffeine (0.5 mg/mL), and NaCl (0.5 M). The cells were spotted onto YPD agar medium containing 0.03% SDS and 0.2% Congo red to assess integrity of cell wall. The spotted cells were incubated at 30 °C for 48 h and imaged. For temperature stress tests, the YPD plates were incubated at both 30 and 37 °C.

### RNA-Seq and differential expression analysis

Each well in a 6-well plate was filled with 3 mL YPD, with (I-H99-1, 2 and 3) or without IBC treatment (H99-1, 2 and 3), and inoculated with a final concentration of 10^6^ cells/mL from a fresh overnight culture of *C. neoformans* H99. The plate assay was conducted for 4 h at 30 °C, after which the cells were pelleted, flash frozen on dry ice, and lyophilized overnight. RNA was extracted from harvested cells and sequenced, following a slightly modified version of a previously described method [41]. The Qiagen RNeasy Plant Mini Kit was used to isolate total RNA from harvested cells, with on-column DNase treatment, as per the manufacturer’s instructions. The quantity and quality of RNA were evaluated using the Agilent 2,100. The NEBNext UltraTM II directional RNA library prep kit for Illumina was applied to construct sequencing libraries (New England Biolabs, Ipswich, MA). These libraries were submitted to the Shanghai Meiji Biomedical Technology (Shanghai, China) for sequencing on the Illumina 6000 platform, which produced 150 bp, single-end reads. The read files were mapped to the *C. neoformans* H99 reference H99 v48 (FungiDB) using the STAR alignment software [42], and gene transcript expression was quantified using HISAT2 and Stringtie. Differentially expressed genes (DEGs) analyses for multiple comparisons were performed in R using a Bioconductor workflow, followed by the DESeq2 package with a false discovery rate (FDR) of 5%. Principle component analysis was carried out on regularized log transformed gene counts to confirm the absence of batch effects [43]. DEGs annotation was performed using Gene Ontology (GO) and Kyoto Encyclopedia of Genes and Genomes (KEGG) databases. GO and KEGG enrichments were carried out using the BioConductor software CLUSTER Profiler 3.4.4 to annotate the function of DEGs.

### Real-time quantitative reverse transcription PCR (RT-qPCR)

The impact of IBC on genes associated with cell wall/membrane, drug resistance, virulence and apoptosis, such as Cat3, Mar1, Erg11, Erg6, Kre5, Kre6, Lac1, Afr1, Pdr6, Mca1 and others, was investigated using RT-qPCR analysis. Initially, total RNA was extracted from *C. neoformans* H99 cells, both in the presence or absence of IBC, using the TRIzol method. This RNA was reverse transcribed into cDNA using a high-capacity cDNA reverse transcription kit (Applied Biosystems). Following this, RT-qPCR analysis was conducted using an SLAN 96 S real-time PCR instrument from Hongshi Medical Technology, Shanghai, China. The Power SYBR green PCR master mix (Applied Biosystems) was used for this process, following the manufacturer’s instructions. The expression values of the candidate DEG gene were determined using the 2^−ΔΔ*CT*^ method in RT-qPCR analysis. All primer sequences for the target genes are provided in Table S1.

### Identification of hub genes and their protein interaction with IBC

Genes from the four key modules were grouped together to construct the heat map based on their intramodular connectivity and function. The genes from each heatmap were used to identify the node hub genes within each heatmap using STRING (Search Tool for the Retrieval of Interacting Genes) [44] and networkX in Python [45]. The node hub gene was selected as the potential target of IBC. The interactions between IBC and potential target proteins were examined following a previously described method. Briefly, the structures of potential target proteins, including the UDP-glucose: glycoprotein glucosyltransferase (KRE6), β-glucan synthesis-associated protein (SKN1), the enzyme P450 lanosterol 14α-demethylase (ERG11), ATP-binding cassette transporter (AFR1), the dynamin-related protein (DNM1), metacaspases (MCA1), the capsule-inducing transcriptional regulator (GAT201), and the homeobox transcription factor (HOB1), were downloaded from the PDB database (https://www.rcsb.org/). The structure file for IBC was downloaded from PubChem (https://pubchem.ncbi.nlm.nih.gov/). Chem3D software was used to perform calculations of molecular mechanics on the optimal conformation of IBC, resulting in the optimal conformation of IBC with minimal energy. AutoDock Tools 1.5.6 was used to hydrotreate the target proteins and IBC, and determine the reversible bonds. POCASA was used to predict the activity pockets of target proteins. Finally, Auto Dock Vina v.1.2.0 software was used to perform molecular simulation docking of target proteins and IBC. The Lamarckian genetic algorithm was used for protein-drug flexible docking, and the docking calculation was semi-flexible docking. After the molecular docking was completed, PyMOL2.3.0 was employed to visualize the docking results.

### *Caenorhabditis elegans* lifespan assay

A model of *C. neoformans* H99 infection in *C. elegans* was developed to assess the in vivo antifungal activity of IBC, following a method previously reported by Pre-laboratory programmes [46]. Briefly, *C. elegans* worms were cultivated on nematode growth medium (NGM) agar plates that had been seeded with *E. coli* TOP50, and then synchronised. The synchronized worms were rinsed with M9 buffer, moved to fresh *C. neoformans* H99 lawns, and inoculated for an additional 6 h at 22 °C. The *C. neoformans*-infected worms were gently washed twice with M9 buffer, moved to NGM plates supplemented with various concentrations of IBC (0 MIC, 1 MIC and 2 MIC), and incubated for 24 h. The worms were collected by washing the plates with M9 buffer. Thirty worms were randomly selected and transferred to each well of a 24-well plate containing NGM liquid medium, and then grown for 25 days at 22 °C. Live worms that were alive and responded to a gentle touch with a sterilized platinum wire were counted every two days, and survival curves were then generated. All experiments were performed in triplicates.

### Statistical analysis

The data collected in this study was analyzed using two software tools: GraphPad Prism 9 software and Microsoft Excel. These tools were chosen for their robust capabilities in handling and interpreting complex data sets. GraphPad Prism 9, a comprehensive statistical and scientific 2D graphing software, was utilized to perform various statistical analyses. This software is renowned for its ability to simplify complex data organization, curve fitting, and scientific graphing, making it an ideal choice for this study. On the other hand, Microsoft Excel, was used for its versatility in data manipulation and analysis. The results were represented visually in the form of graphs. The descriptions and statistical analyses related to these graphs were provided in the figure legends, offering a detailed explanation of each figure and its relevance to the study.

## Results and discussions

### MIC of IBC against *C. Neoformans* H99

The MIC of IBC, when tested against *C. neoformans* H99, was found to be 8 µg/mL. The impact of IBC on the growth kinetics of *C. neoformans* H99 was elucidated by measuring the optical density (OD) value at 600 nm throughout the cultivation process. Fig. S1 demonstrates that, IBC exhibited an obvious inhibitory effect on the growth of *C. neoformans* H99 during a 24-h period, indicating that IBC at 8 µg/mL showed strong antifungal activity against *C. neoformans* H99 in comparison with the control group. Findings from past studies has revealed that *C. neoformans* is a serious public health threat due to its capability to cause disease in the immunocompromised individuals and the emergence of antifungal resistance among *Cryptococcus* species. In addition, an increasing number of biofilm-associated infections by *C. neoformans* were becoming progressively more challenging to treat by conventional antifungal treatments [47]. Therefore, the focus on antifungal and antibiofilm therapy-based research has dramatically increased in recent years. In the present study, the antifungal and antibiofilm efficacy of IBC was explored by inhibiting the growth, biofilm formation and virulence factor production of *C. neoformans*.

#### IBC induced cell wall/membrane damage and decreased metabolic activity in *C. Neoformans* H99

**Fig. 1:**
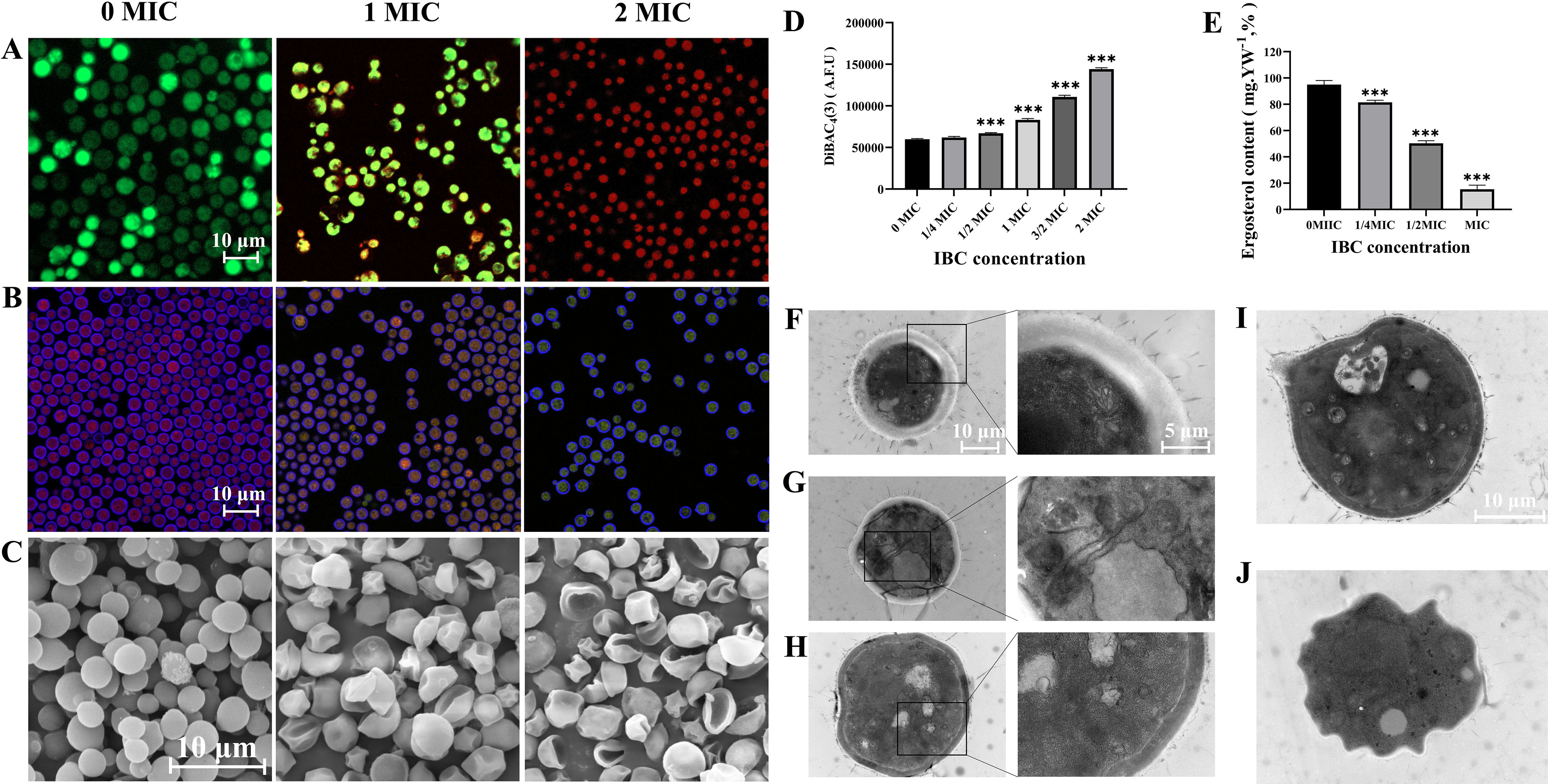
IBC affected the cell membrane integrity (**A**), metabolic activity (**B**) and cell morphology (**C**) of *C. neoformans*. CLSM images of *C. neoformans* cells in the presence of various concentrations of IBC (0 MIC, 1 MIC and 2 MIC) were obtained using SYTO9/PI (A), and FUN® 1/CWS (B) double dyes, respectively. Furthermore, *C. neoformans* treated with various concentrations of IBC at 30 °C for 6 h was investigated using FESEM. The scale bar for CLSM and FESEM images was 10 μm. IBC altered cytoplasmic membrane potential (**D**), decreased the ergosterol content (**E**) and disrupted the fungal ultrastructure (F-J) in *C. neoformans.* Quantitative analysis of cytoplasmic membrane potential and ergosterol contents was performed in IBC-treated *C. neoformans* cells. In addition, cells were viewed under a TEM microscope, in which fungal cells were incubated in the absence (**F**, **G**) and presence (**H**-**J**) of IBC at 1 MIC for 24 h. Scale bar was 1 μm for the TEM images, and 500 nm for the extended images. Bars represent the standard deviation (*n* = 3). *p < 0.05; **p < 0.01; ***p < 0.001

The integrity of cell membrane was qualitatively evaluated using SYTO 9 and PI double staining. Significant difference was observed in the integrity of cell membrane between the control group and high-dose IBC-treated group, as shown in Fig. 1A, The control group produced clear bright green fluorescence, while the ratio of red/green fluorescence increased with the concentration of IBC, indicating an increase in the number of *C. neoformans* H99 cells with damaged cell membrane integrity. The metabolic activity of fungal cells was assessed using FUN ® 1 and CWS double staining. FUN ® 1 differentiates between metabolically active and metabolically inactive or dead fungal cells. The red-fluorescence was produced for the cylindrical intravacuolar structures of metabolically active cells, which was diffused, and the yellow-green fluorescence was established for metabolically inactive or dead fungal cells. As shown in Fig. 1B, it was almost non-existent to observe the transition from red fluorescence to yellow-green fluorescence in untreated group. However, when exposed to 1 MIC or 2 MIC concentrations of IBC, the proportion of red fluorescence significantly decreased, indicating a decrease in the number of *C. neoformans* H99 cells red fluorescence with the increasing IBC concentration. Furthermore, the more noticeable effect in the cell morphology was observed in fungal cells treated with IBC at 4 µg/mL or 8 µg/mL. FESEM analysis revealed a smooth compact structure of the untreated cell of *C. neoformans* H99, whereas IBC treated cells showed deformities of cell morphology (Fig. 1C), as evidenced by the extensive wrinkles on the cell surfaces of *C. neoformans* H99. A rise in the amount of *C. neoformans* H99 cells with damaged plasma membrane integrity and compromised cellular metabolic activity was observed in CLSM images of IBC-treated samples, which validated the antifungal potential of IBC against *C. neoformans* H99 by altering cell membrane permeability.

#### IBC altered cytoplasmic membrane potential, decreased the ergosterol content and disrupted the fungal ultrastructure in ***C. neoformans*** H99

The potential impact of IBC on the properties of fungal membrane was examined by measuring alterations in cytoplasmic membrane potential using DiBAC_4_(3). An increase in fluorescence of DiBAC_4_(3) indicates membrane depolarization, as DiBAC_4_(3) can enter depolarized cells where it binds to intracellular components and exhibits enhanced fluorescence [48]. As evidenced in Fig. 1D, treatment with IBC at 1/2 MIC or higher resulted in a significant difference in the fluorescence intensity compared to the untreated group, with an increase in the fluorescence intensity observed in cells treated with IBC. Notably, in fungal cells treated with 1/2 MIC or 1 MIC IBC, the fluorescence intensity of DiBAC_4_(3) increased by 11.9% and 38.86%, respectively, compared to untreated cells (Fig. 1D), correlating with the dosage of IBC. The mechanistic results indicated that IBC induced cytoplasmic membrane potential dissipation in the *C. neoformans*. Moreover, a decrease in the ergosterol content of plasma membrane was observed for IBC treated cells in a dose-dependent manner. In the *C. neoformans* cells treated with IBC at 4 or 8 µg/mL, the reductions in ergosterol content were 47.08% and 83.85%, respectively, compared to the control cells (Fig. 1E). Ergosterol, the most abundant sterol in fungal membranes, is critical for regulating membrane permeability and fluidity, similar to mammalian cholesterol, and hence, the influence of IBC on ergosterol production was evaluated, and a low abundance of ergosterol in IBC-treated samples was observed, indicating that IBC may have an effect on ergosterol production [49]. Additionally, the altered membrane potential induced by IBC in treated samples was clearly observed with DiBAC_4_(3), as evidenced by a rise in the membrane potentials in the IBC-treated samples [50]. This data revealed that IBC substantially altered the plasma membrane integrity of *C. neoformans* [51]. TEM analysis showed that normal and intact plasma membranes in untreated fungal cells, and the cell wall was in close contact and there were no changes in the nucleus, vacuoles, mitochondria, cytoplasm, or cell wall (Fig. 1F, G). By contrast, the IBC-treated fungal cells showed profound changes in cell wall/membrane and cell components, and a thinner cell wall (Fig. 1H-J). IBC treated cells exhibited a thinner cell wall, alterations in the space between cell wall and plasma membrane, an increase in the number and size of the vacuoles (Fig. 1I), and a complete loss of the normal fungal cell form (Fig. 1J). In this study, microscopy analyses such as CSLM and TEM assays suggested that IBC at 8 µg/ml caused loss of plasma membrane integrity of *C. neoformans* H99. These qualitative and quantitative analysis results further indicate that IBC exerts antifungal effects by influencing cell membrane and cell wall.

#### Effect of IBC on *C. Neoformans* H99 biofilm formation and mature biofilm

**Fig. 2:**
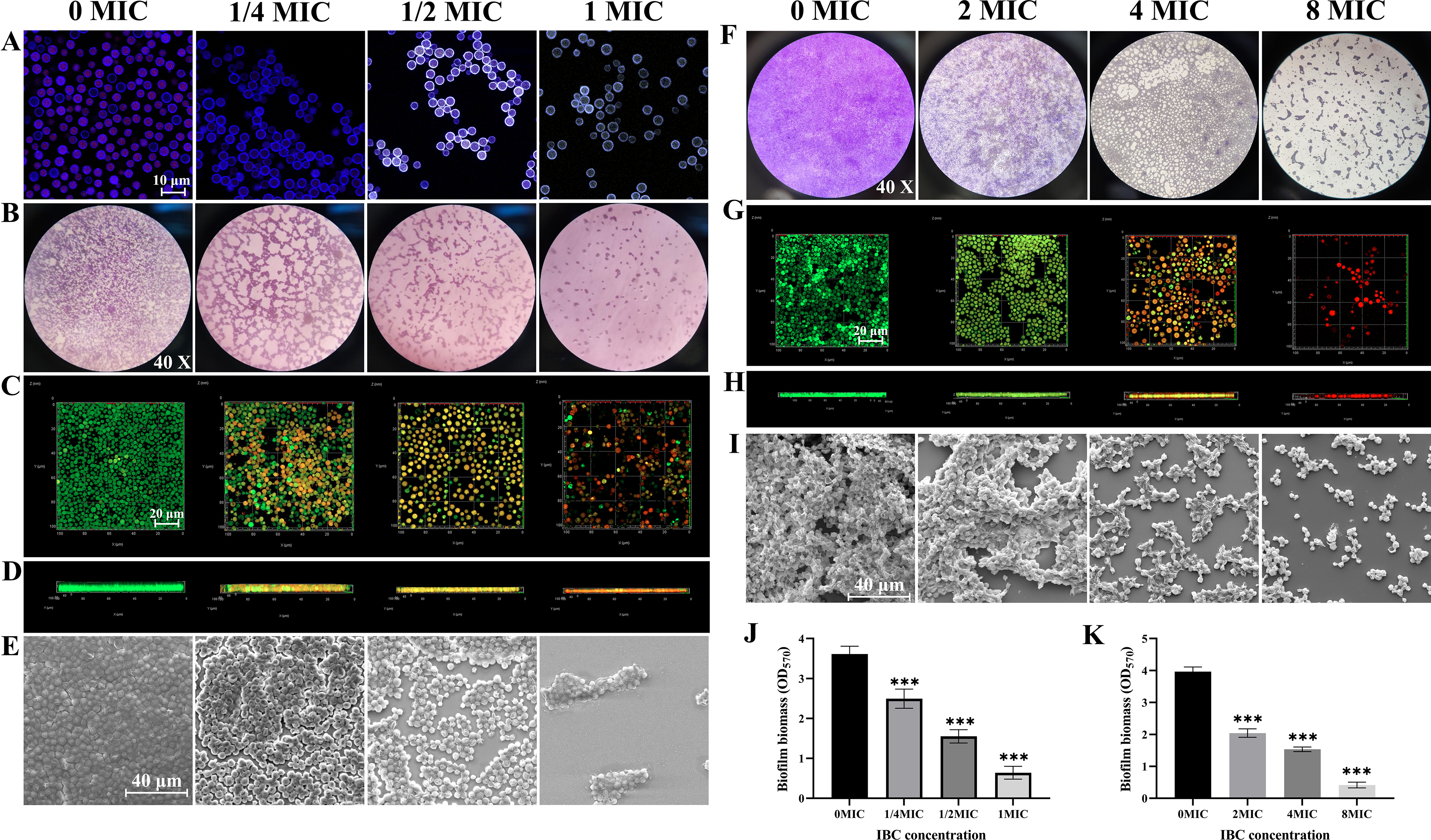
IBC showed the inhibitory effect on *C. neoformans* biofilm formation by inhibiting initial attachment and maturation. (**A**) The anti-adhesion ability of IBC to the glass surface was examined using FUN® 1/CWS double dyes combined with CLSM. (**B**-**E**) The inhibitory effect of IBC on the biofilm formation by *C. neoformans* was performed using CV staining (B, objective, × 40), CLSM (C, plane images; D, three-dimensional images) and FESEM (**E**), respectively. Scale bar was 10 μm for CLSM and 40 μm for FESEM, respectively. IBC exhibited the dispersal effect on mature biofilms by *C. neoformans*. Examination of the ability of high concentrations of IBC to disperse/kill 48-h mature biofilms by *C. neoformans* was carried out. The dispersal/killing effect of IBC on 48-h mature biofilms was evaluated using CV staining (**F**, objective, × 40), CLSM (**G**, plane images; **H**, three-dimensional images) and FESEM (**I**), respectively. (**J**-**K**) The inhibitory and dispersal effect of IBC on biofilm formation and preformed biofilms were quantitatively assessed using CV staining, respectively. Scale bar was 10 μm for CLSM and 40 μm for FESEM, respectively. Bars represent the standard deviation (*n* = 3). *p < 0.05; **p < 0.01; ***p < 0.001

The impact of IBC on the adhesion of *C. neoformans* H99 to glass slides surfaces in a 24-well plate was assessed. Microscopic analysis revealed that IBC at 4 µg/mL effectively inhibited the adhesion of *C. neoformans* H99 to slides surfaces, reduced the number of metabolically active *C. neoformans* H99 cells, and cells exhibiting green fluorescence were observed (Fig. 2A). Furthermore, the influence of IBC on biofilm formation by *C. neoformans* H99 on the glass slides surfaces was examined. IBC reduced biofilm biomass, volumes and structure in a dose-dependent manner (Fig. 2B-D). Light microscopy results clearly demonstrated that there was a dose-dependent reduction in the surface area of biofilm covered on the glass slide in the IBC treatment group in the control group (Fig. 2B). At the 1/2 MIC and 1 MIC IBC-treated group, the biofilm biomass was reduced by 57.06% and 82.54% compared to the control group, respectively (Fig. 2J). CLSM images validated the formation of multilayered biofilms in the control group, while the IBC-treated group exhibited the formation of thinner and dispersed biofilms (Fig. 2C, D). CLSM images also showed that IBC at 2 µg/mL or higher compromised some cells within the biofilm (Fig. 2D). Furthermore, FESEM images of the biofilm in the untreated group displayed thick and closely aggregated cell lining on the glass slides surface, whereas a reduction in extracellular matrix and a disintegrated biofilm were observed in the IBC-treated group (Fig. 2E). Altogether, the microscopic analyses demonstrated that IBC at sub-MIC concentrations was efficacious in inhibiting growth of new biofilm *C. neoformans* H99. Light microscopic, CLSM and FESEM analyses were conducted to investigate the effect of IBC on the biofilm by *C. neoformans* H99. In the presence of 1/2MIC IBC, the substantial reduction in the biofilm coverage area on the surface and the biofilm thickness in IBC-treated samples compared to the untreated control were observed by inhibiting adhesion and biofilm formation of *C. neoformans* H99 to glass surface.

Next, microscopic analyses were conducted to assess the effectiveness of IBC against mature biofilms of *C. neoformans* H99. The light microscopy images revealed that IBC was effective in treating entrenched mature biofilm of *C. neoformans* H99 in a dose-dependent manner, compared to the untreated control (Fig. 2F). CSLM combined with fluorescent staining showed a decrease in micro colonies attached to the abiotic surface, with complex biofilm architecture invisible. IBC at 4 MIC or 8 MIC compromised the cells within the mature biofilm (Fig. 2G, H). Furthermore, FESEM images revealed a significant increase in the number of biofilm-disassembled cells as small multicellular aggregates in the group treated with 4 MIC IBC. In contrast, the structure of mature biofilms in the untreated sample exhibited a compact three-dimensional tower structure (Fig. 2I). At the 4 MIC and 8 MIC IBC-treated group, the mature biofilms were dispersed by 61.36% and 89.65% compared to the control group, respectively (Fig. 2K). Previous studies have confirmed that biofilm-producing pathogens are generally much less sensitive to antimicrobials compared to their planktonic counterparts [16, 17]. Moreover, antibiotics have limited dispersal effects on mature biofilms, while IBC demonstrates good dispersal capabilities on mature biofilms. This implies that IBC can be used alone or in combination with antibiotics for antibiofilm activity against mature biofilms.

#### IBC altered mitochondrial morphologies and induced mitochondrial dysfunction

**Fig. 3:**
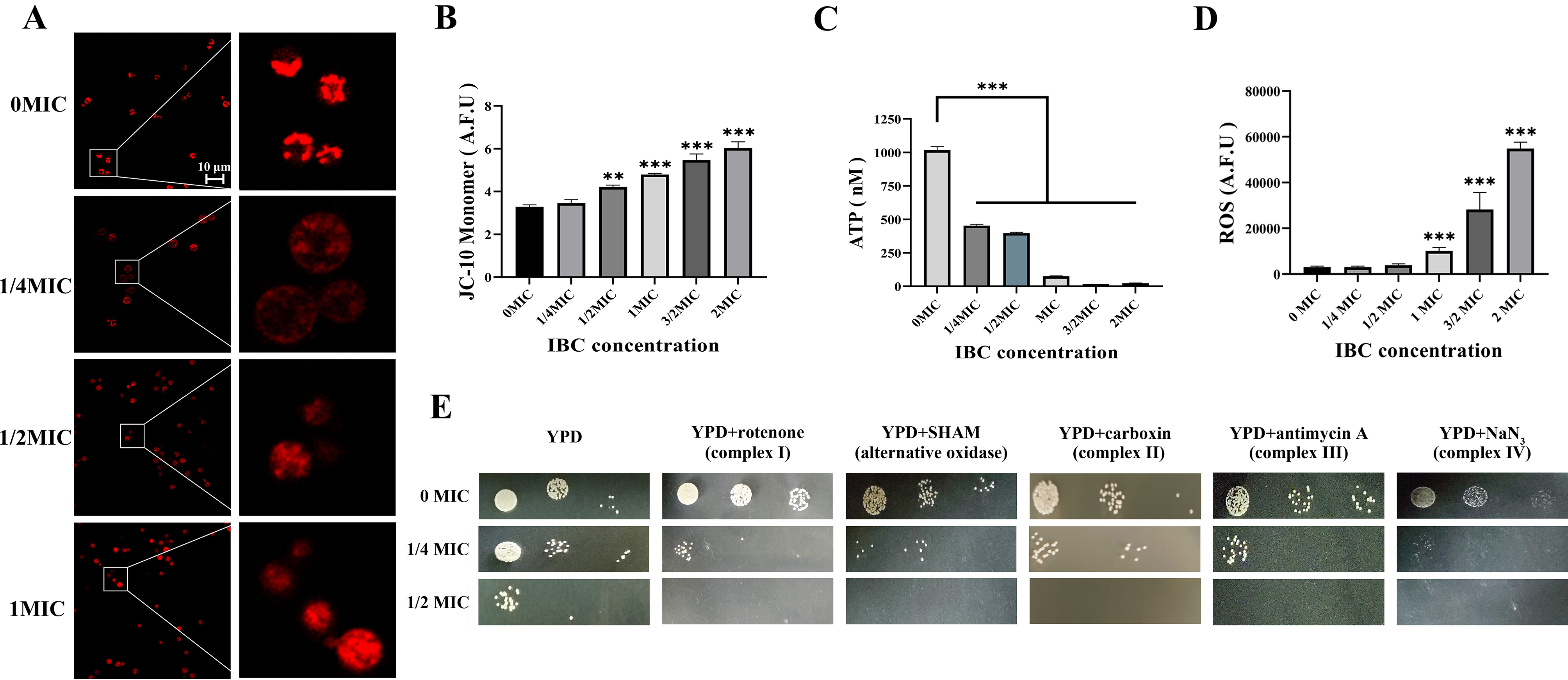
IBC altered mitochondrial morphologies and induced mitochondrial dysfunction of *C. neoformans.* (**A**) Mitochondrial morphologies of *C. neoformans* were assessed following incubation in YPD medium supplemented with various concentrations of IBC at 30 °C. MitoTracker Red CMXRos was used to stain diffuse, tubular, or fragmented morphologies, and mitochondria were then imaged using CLSM. The right images are the enlarged image of the white square on the left images. (**B**) MMP of *C. neoformans* was measured following incubation in YPD medium supplemented with various concentrations of IBC at 30 °C. Mitochondria were stained with JC-10 marker of MMP. The fluorescence microplate reader was used to quantify staining intensity. Experiment was repeated three times to ensure reproducibility. (**C**) Total intracellular ATP was measured for the *C. neoformans* following incubation in YPD medium supplemented with various concentrations of IBC at 30 °C. Normalized total cell lysates were determined for total intracellular ATP using a firefly luciferase bioluminescent assay. (**D**) Total intracellular ROS was measured for *C. neoformans* following incubation in the absence and presence of various concentrations of IBC. Cells were stained with DCFH-DA as a marker for intracellular ROS. The fluorescence microplate reader was used to quantify staining intensity. (**E**) IBC affects the electron transport chain function. Growth phenotypes of *C. neoformans* were assessed in the presence of electron transport chain inhibitors. Cells were normalized by OD_600_ and subsequently cultured on YPD medium agar plates containing rotenone (0.5 mg/mL), salicylhydroxamic acid (SHAM) (2.5 mM), carboxin (50 µg/mL), antimycin A (3 µg/mL), and sodium azide (NaN_3_) (0.5 mM) at 30 °C, respectively. Plates were imaged daily. Experiment was repeated two times to ensure reproducibility and representative images are shown

Mitochondrial morphology was assessed using MitoTracker Red CMXRos staining. As shown in Fig. 3A, consistent with previously reported observations, three mitochondrial morphologies were observed: diffuse, tubular, and fragmented [31]. In YPD medium incubated at 30 °C, most cells across all strain displayed a tubular morphology. Cells treated with 1/2MIC IBC displayed an increase in the percentage of cells with diffuse morphologies and a corresponding decrease in cells with tubular or fragmented morphology (Fig. 3A). These morphological changes were anticipated as the mitochondria undergo fusion and fission in response to IBC treatment, resulting in the changes of mitochondrial morphologies, respectively.

Changes in the MMP and ATP production of *C. neoformans* H99 cells in the presence or absence of IBC were evaluated using the specific fluorescent probe JC-10 and firefly luciferase bioluminescent assay, respectively. Treatment with IBC resulted in a concentration-dependent decrease in MMP and ATP production of *C. neoformans* H99. A significant difference in MMP and ATP yield was observed between samples treated with 1 MIC or higher and the untreated sample (Fig. 3B, C). Moreover, ROS accumulation was evaluated using DCFH-DA in *C. neoformans* H99 treated with or without IBC. IBC treatment increased the ROS generation in a concentration-dependent manner, and a significant difference observed between samples treated with 1 MIC or higher and the untreated samples (Fig. 3D).

Mitochondria are called “power stations” because it plays an important role in aerobic respiration which provides energy to fuel all cellular processes [52]. In addition to generating the energy necessary to power cells, mitochondria also play an important role in coordinating other cell processes such as apoptosis, and Ca^2+^ signal transduction [53]. Similarly, previous studies have demonstrated the importance of mitochondrial morphology for fitness and virulence in *Cryptococcus* species [31]. CLSM analysis observed the increase in diffuse morphology in IBC-treated *C. neoformans* H99 cells, and the decrease in tubular and fragmented morphologies. FESEM and TEM analysis revealed alterations in the cell morphology with collapsed cell surfaces and disrupted organelles in IBC-treated samples. Furthermore, visualizing changes in the mitochondrial morphology of *C. neoformans* H99 in the IBC-treated samples were evaluated using mitochondrial-specific fluorescent dyes. IBC treatment resulted in the mitochondrial dysfunction, with aberrant mitochondrial morphology, decreased ATP level, and decreased MMP as a result of the elevation of IBC. Meanwhile, the increase in ROS level was observed, which could disturb mitochondrial quality, and the resultant dysfunctional mitochondria were fragmented. In addition, previous studies have shown that the inhibition of electron transport chain function can exert the mitochondrial toxicity effect [54, 55]. To examine this function, the effect of IBC on the electron transport chain of *C. neoformans* H99 was assessed, and the data revealed that IBC significantly affected the electron transport chain of *C. neoformans* H99. These effects, in combination with the inhibitory effect on the mitochondrial morphology and function may explain the phenomenon that severe mitochondrial damage caused by IBC, which leads to *C. neoformans* H99 cell death [56].

The effect of IBC on oxidative phosphorylation was explored, and the ability of *C. neoformans* H99 to grow on YPD medium containing electron transport chain inhibitors was evaluated. As shown in Fig. 3E, C. *neoformans* H99 demonstrated significantly impeded growth in the presence of inhibitors of complex I (rotenone), the alternative oxidase (SHAM), complex III (antimycin A), and complex IV (NaN_3_) at 1/2 MIC IBC and 1/4 MIC IBC, as compared to YPD medium alone, indicating that the growth inhibition was due to the IBC activity. Altogether, the treatment with IBC caused a decreased MMP and a reduction in ATP production, and induced ROS accumulation. These results further suggests that the antifungal activity of IBC involves altering the morphology and metabolic activity of mitochondria.

**IBC improved the** ***C. neoformans*** **H99 susceptibility to fluconazole and cell stressors and decreased virulence factor**

**Fig. 4:**
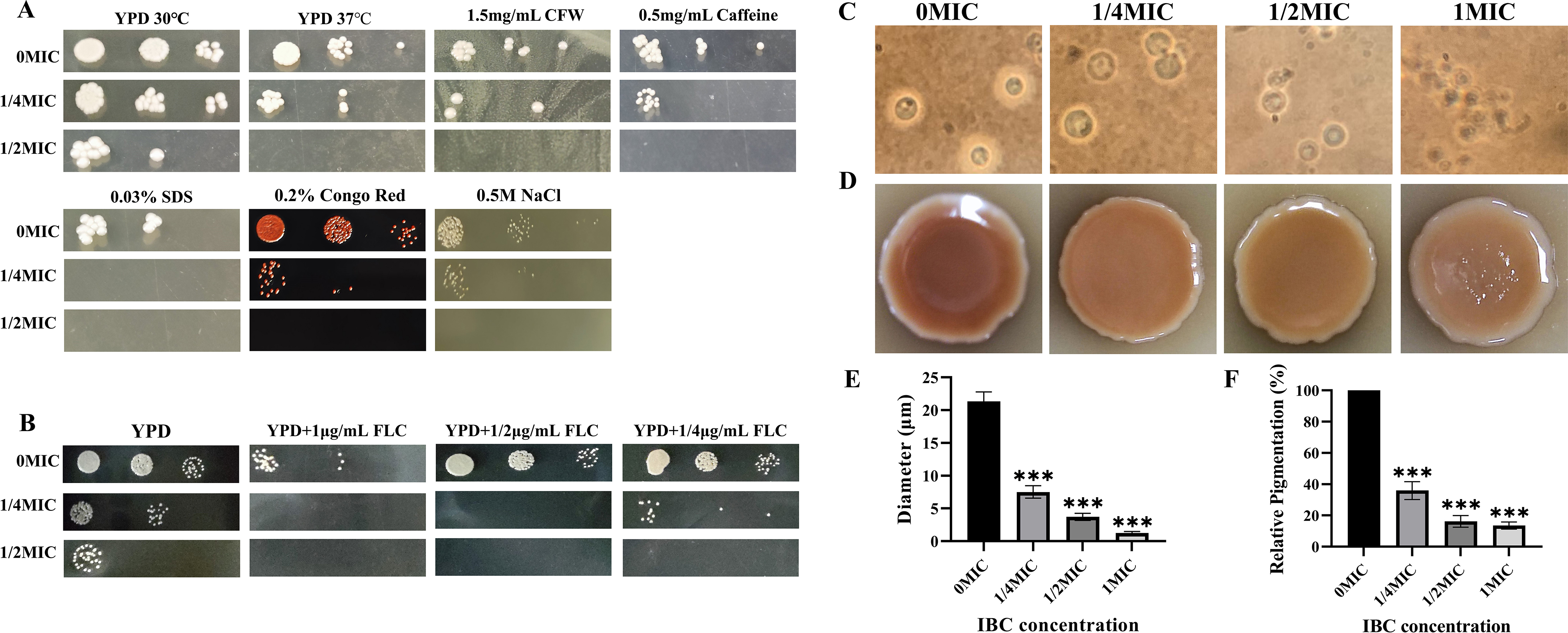
IBC improved the susceptibility of *C. neoformans* to cell stressors and fluconazole and decreased virulence factor production. (**A**) Growth phenotypes of *C. neoformans* were assessed in the presence of various cell stressors and different concentrations of IBC. Cells were normalized by OD_600_ and subsequently diluted. 5 µL aliquots of serial dilutions were subsequently spotted on YPD agar medium supplemented with calcofluor white (1.5 mg/mL), caffeine (0.5 mg/mL), and NaCl (0.5 M), 0.03% SDS and 0.2% Congo red in the presence of various concentrations of IBC at 30 °C, respectively. For temperature stress, the YPD plates were incubated at 37 °C in the presence of various concentrations of IBC. Experiment was repeated three times to ensure reproducibility and representative images are shown. (**B**) Fluconazole susceptibility was assessed for the IBC-treated *C. neoformans* cells. Cells were normalized by OD_600_ and were subsequently grown on YPD medium agar plates in the presence of fluconazole and different concentrations of IBC. Cells were incubated at 30 °C and plates were imaged at the indicated times. (**C**, **E**) Representative images of various concentrations of IBC-treated *C. neoformans* grown in Dulbecco’s Modified Eagle’s Medium (DMEM) for 48 h at 37 °C. Capsules were visualized by India ink staining and examined under a microscope, and the capsule diameter was evaluated. (**D**, **F**) *C. neoformans* cells were grown on L-DOPA agar plates for 72 h at 30 °C with or without the addition of IBC. Reduced amounts of melanin formation in the colonies were observed in a concentration-dependent manner

The sensitivity of *C. neoformans* H99 to various cell stressors was examined, and *C. neoformans* H99 was cultured on YPD agar media supplemented with various cell stressors and various concentrations of IBC. As shown in Fig. 4A, C. *neoformans* H99 showed sensitivity to six cell stressors tested in the presence of 1/2 MIC and 1/4 MIC IBC, and at 1/2 MIC IBC, *C. neoformans* H99 failed to grow in the presence of six cell stressors tested. Notably, at 1/4 MIC IBC, 0.03% SDS entirely inhibited the growth of *C. neoformans* H99, and 0.5 mg/mL caffeine resulted in *C. neoformans* H99 colonies of tiny size.

Considering aberrant oxidative stress widely triggers abnormalities of organelle functions, the alterations in the susceptibility of the IBC-treated *C. neoformans* H99 cells to fluconazole were hypothesized to induce oxidative stress. After 48 h of incubation in the presence of a IBC gradient, IBC remarkably enhanced the sensitivity of fluconazole against *C. neoformans* H99 with a MIC of 0.25 µg/mL fluconazole and 2 µg/mL IBC (Fig. 4B). Moreover, the fungicidal activity of fluconazole was found to be enhanced in the presence of IBC in a dose-dependent manner. These results clearly indicate that IBC increased the susceptibility of *C. neoformans* H99 to fluconazole.

In addition to growth at 37 °C, the fungal pathogen *C. neoformans* H99 has two major virulence factors: capsule synthesis, and melanin formation. When treated with India Ink exclusion staining, capsules were imaged under a microscope. The results from this experiment revealed a remarkable decrease in capsule synthesis in samples treated with 1/2 MIC or 1 MIC IBC treated samples (Fig. 4C). Additionally, at the 1/4 MIC and 1/2 MIC IBC-treated group, the capsule diameter of *C. neoformans* H99 was reduced by 64.77% and 82.56% compared to the control group, respectively (Fig. 4E). Similarly, as expected, IBC-treated cells showed a reduction in melanin formation in a concentration-dependent manner (Fig. 4D). Moreover, at the 1/4 MIC and 1/2 MIC IBC-treated group, the relative pigmentation of *C. neoformans* H99 was reduced by 64.07% and 83.81% compared to the control group, respectively (Fig. 4F). The above results suggest that the antifungal mechanism of IBC could be multifaceted, involving the modulation of fungal growth, metabolism, and the production of virulence factors.

### Involvement of apoptosis in IBC-induced *C. Neoformans* H99 cell death

**Fig. 5:**
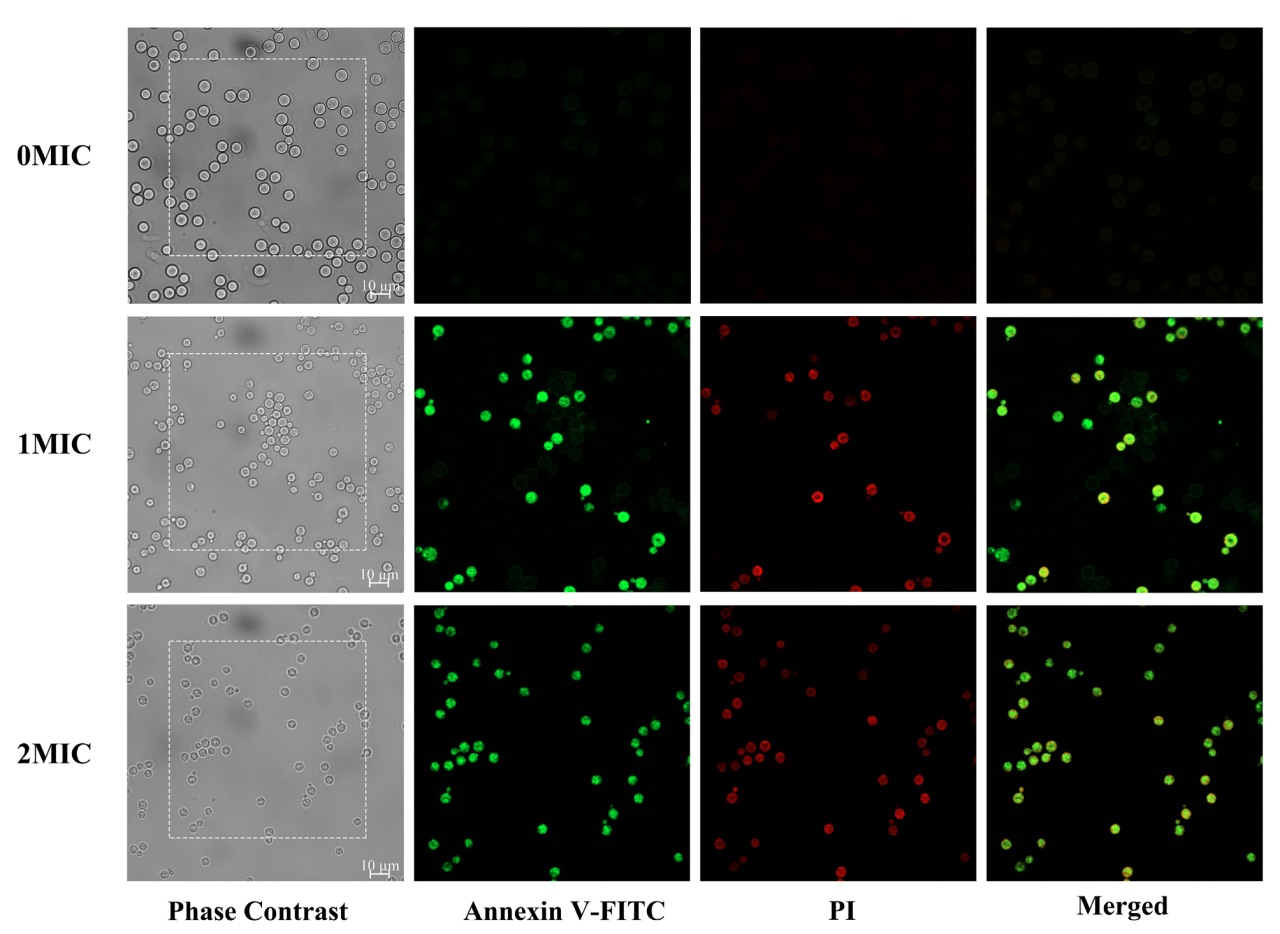
Verification of apoptotic and necrotic cell death using standard methods. CLSM analysis of cells were stained using Annexin V/PI dyes after exposure to different concentrations of IBC.

The study investigated the induction of apoptosis in *C. neoformans* H99 by IBC. The method used for this examination was CLSM with Annexin V-FITC/PI double staining, applied after treating the cells various concentrations of IBC. As displayed in Fig. 5, early apoptotic cells (Annexin V+/PI−) were detected when treated with 1MIC IBC. However, the number of late apoptotic cells or necrotic cells (Annexin V+/PI+) significantly enhanced when concentration was raised to 2 MIC IBC. On the other hand, the untreated group showed healthy viable (Annexin V−/PI−) cells, suggesting that IBC triggered apoptosis in *C. neoformans* H99 in a concentration-dependent manner. The role of apoptosis-inducing factor (Aif1) and metacaspases (Mca1 and Mca2) in apoptosis-like cell death in *C. neoformans* H99 was observed in a study. The disruption of Aif1 was found to stimulate the emergence of aneuploid subpopulations that were resistant to fluconazole in vitro and in vivo. This suggested that apoptosis orchestrated by Aif1 might eliminate aneuploid cells from the population and defects in this pathway contributed to the selection of aneuploid fluconazole subpopulations during treatment [57]. In a study, the antifungal potential of synthetic peptides against *C. neoformans* H99 was assessed. The study revealed that these synthetic peptides induced membrane-pore formation, DNA degradation, and apoptosis [58]. These results suggest that IBC may activate fungal apoptosis by mainly affecting Aif1, thereby exerting its antifungal activity.

### Identification of *C. Neoformans* H99 DEGs treated with IBC

**Fig. 6:**
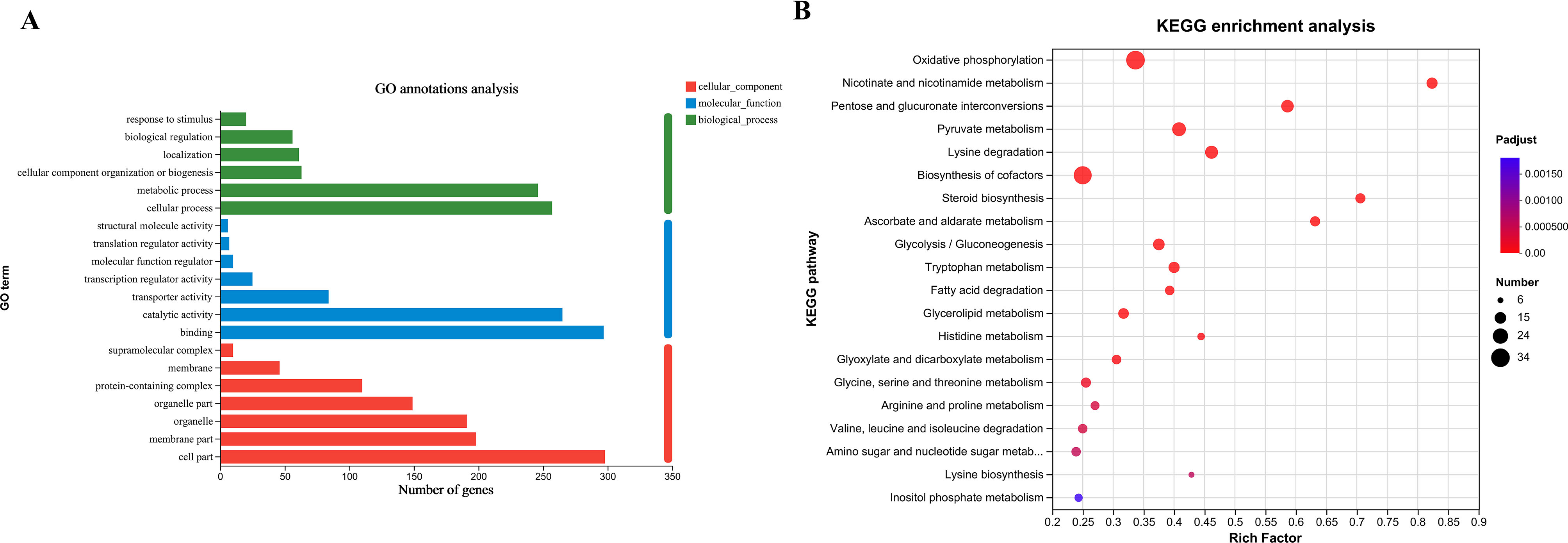
DEGs analysis of untreated and IBC-treated *C. neoformans* cells using RNA-seq analysis. RNA-seq analysis was performed on total RNA extracted from three replicate samples for each biological group. (**A**) GO enrichment analysis of DEGs was performed to identify the most significantly enriched pathways at a threshold Padjust of ≤ 0.05. (**B**) Scatter plot of KEGG enrichment of DEGs. The abscissa Rich factor indicates the number of DEGs located in the KEGG/the total number of genes located in the KEGG metabolic pathway

RNA-seq analysis was performed to examine the intracellular mRNA samples isolated from *C. neoformans* H99 cultured in the absence and presence of 1 MIC IBC. The DEGs were identified in the samples treated with IBC. Fig. S2A shows a high reproducibility of RNA-seq data among biological replicates and between the different samples. The volcano map in Fig. S2B marks up- and downregulated genes with green and red dots, respectively. It shows that 253 genes were upregulated and 865 genes were downregulated in the IBC-treated group compared to the untreated group. The GO analysis revealed that DEGs were primarily enriched in five biological processes: metabolic processes, cellular processes, catalytic activity, binding, membrane part and cell part. The KEGG analysis further indicated that the DEGs were mainly enriched in five biological pathways, including oxidative phosphorylation, pentose and glucuronate interconversions, pyruvate metabolism, lysine degradation and biosynthesis of cofactors. These pathways were mainly related to the cell wall, cell membrane, drug resistance, virulence and apoptosis related pathways (Fig. 6A-B).

**Fig. 7:**
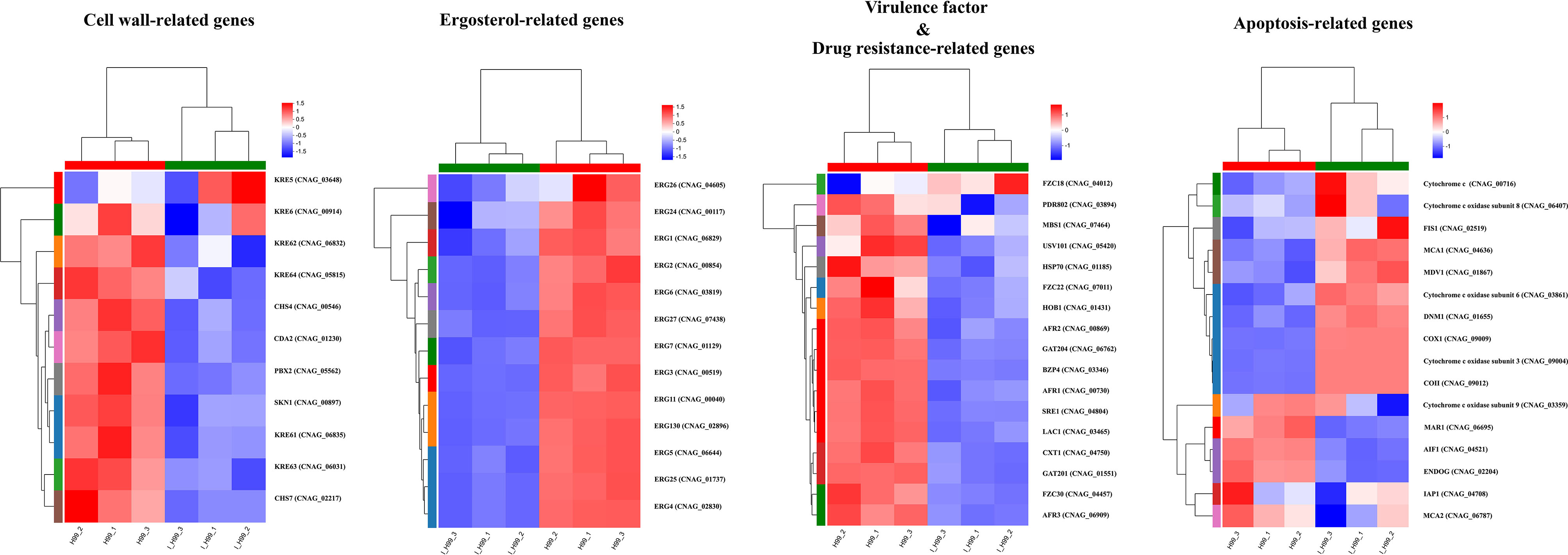
Gene set enrichment analysis plot depicting the enrichment of DEGs in IBC-treated and untreated *C. neoformans* cells. The expression pattern of candidate DEGs related to cell wall synthesis in IBC-treated *C. neoformans* cells. The expression pattern of candidate DEGs related to the ergosterol synthesis in IBC-treated *C. neoformans* cells. The expression pattern of candidate DEGs pertaining to drug efflux in IBC-treated *C. neoformans* cells. The expression pattern of candidate DEGs involved in the maintenance of mitochondrial homeostasis in IBC-treated *C. neoformans* cells. The expression pattern of candidate DEGs involved in the melanin and capsule biosynthesis in IBC-treated *C. neoformans* cells

In the fungal cell wall pathway, IBC downregulated several cell wall related-genes (KRE5, KRE6, KRE61, KRE62, KRE63, KRE64, SKN1, CHS4, CHS7 and CDA2) which are necessary for cell wall maintenance (Fig. 7). Among them, KRE family genes (KRE5, KRE6, KRE61, KRE62, KRE63, and KRE64) have been identified and characterized by putatively involved in β-1,6-glucan synthesis, and cell wall protein anchoring in *C. neoformans*. The KRE5, KRE6 and SKN1 were demonstrated as critical for maintaining growth, morphology and cell wall integrity in *C. neoformans* [59]. Similarly, CHS4, CHS7 and CDA2 are putatively involved in chitin, which is responsible for the rigidity of cell wall. In this study, the results of RNA-seq showed the down-regulation of Kre5, kre6, Kre61, Kre62, Kre63, Kre64, and Skn1 genes, which were consistent with results of microscopic examination and cell stress assay [60].

Interestingly, enrichment analyses and fine-mapping with gene expression in IBC-treated and untreated samples implicated the expression of 13 within 25 known ergosterol (ERG) biosynthesis genes, which was downregulated in response to IBC treatment, including key ERG3, ERG6, ERG11, and ERG25 (Fig. 7). Ergosterol is considered as critical sterol in the cell membranes of fungi, and a common target of antifungal drug treatment is the fungal ergosterol biosynthesis pathway [61]. Enhanced drug efflux is also a dominant mechanism of resistance to diverse antifungals and involves the upregulation of ATP-binding cassette (ABC) transporters. In *C. neoformans*, overexpression of the ABC transporters AFR1 mediates azole resistance. The effects of IBC on drug resistance-related gene expression were shown in Fig. 7. IBC significantly decreased the expression levels of AFR1, AFR2, and AFR3 [62].

Notably, as shown in Fig. 7, IBC also affected the expression of genes encoding mitochondrial proteins that impacted mitochondria homeostasis. IBC downregulated the expression of MAR1 gene, which supported the proper electron transport chain function and maintained the mitochondrial homeostasis, including mitochondrial mass and MMP [35]. Similarly, IBC upregulated the expression of dynamin-related GTPase (Dnm1), mitochondrial fission protein 1 (Fis1) and mitochondrial division protein 1 (Mdv1) genes, related to mitochondrial morphology. In addition, IBC downregulated the expression of apoptosis-inducing factor (Aif1) gene and upregulated the expression of cytochrome c gene, related to apoptosis [63–66].

In *C. neoformans*, heteroresistance is often acquired through the disomies formation, the most prevalent being that of chromosome 1, which contains both Erg11 and Afr1. Findings inferred that IBC could reverse drug resistance mediated by AFR1 and ERG11 via decreasing their gene expressions, which was consistent with the results of synergistic activity assay of IBC and fluconazole. Furthermore, IBC significantly downregulated the Lac1 gene expression, encoding proteins laccase, which is the dominant producer of melanin in *C. neoformans*. In addition to downregulating Lac1, IBC also significantly downregulated the Hob1, Bzp4, Usv101, and Mbs1 gene expression, encoding four core transcriptional factors in a melanin-regulatory signaling network (Fig. 7). Furthermore, IBC significantly downregulated Gat201 gene expression, and upregulated Fzc18 gene expression. A previous study showed that Bzp4, and Gat201 govern capsule formation in *C. neoformans* by regulating the expression of various capsule biosynthesis genes in a positive manner, whereas Fzc18 as the negative regulator regulated the capsule production (38–39, 67). RNA-seq analysis also revealed that IBC treatment downregulated the expression of Hob1, Bzp4, Usv101, Mbs1, and Lac1. Previous study showed that according to a melanin-regulatory signaling network study, four core transcriptional factors (Hob1, Bzp4, Usv101, and Mbs1) were required for *C. neoformans* melanization, which was mainly regulated by controlling the expression of laccase enzymes, primarily LAC1. BZP4, USV101, and MBS1 independently regulated the LAC1 induction, whereas HOB1 controlled the expression of Bzp4 and Usv101 [38]. This result was consistent with the results of the melanin assay in the present study. The upregulation of plasma membrane efflux pumps is a common mechanism by which cells govern antifungal resistance in major fungal pathogens. The ATP-binding cassette (ABC) superfamily, belonging to the first main class of efflux pumps, implicated in antifungal drug resistance [67]. In *C. neoformans*, the roles of AFR1, AFR2 (also called PDR5), and MDR1 as azole efflux pumps have been well documented, where AFR1 functions as the major pump and AFR2 and MDR1 provided additional roles in the management of fluconazole stress [68]. Consistently, RNA-seq analysis revealed that IBC treatment affected the expression of genes related to mitochondrial morphology, which was further validated by RT-qPCR analysis.

Next, the STRING database was used to perform protein interaction network analysis of the module genes. The protein interaction network was constructed based on the corresponding interaction relationships according to *C. neoformans* database. Then, the networkX in Python was used to visualize the network of genes of interest. And the connectivity of each node in the protein-protein interaction network was statistically analyzed, and the connectivity of nodes in the graph is directly proportional to their size [69]. As shown in Fig. S3, Erg11 (CNAG-0040), Afr1 (CNAG-00730), Dnm1 (CNAG-01655) and Hob1 (CNAG-01431) were identified as the genes of interest.

### Binding mechanisms of IBC to key target proteins of *C. Neoformans* H99

In this study, we constructed protein models for eight potential candidate target proteins, including KRE6, SKN1, ERG11, AFR1, DNM1, MCA1, GAT201, and HOB1 proteins. The binding ability of the small molecule to the target protein was assessed based on the value of the binding free energy. If the value of the free energy is less than − 5 kcal/mol, it indicates the small molecule has a favorable binding ability to the target protein. Generally, the lower the binding free energy is, the more stable the binding conformation will be [70]. Then, the interactions between the IBC and target proteins were examined via molecular docking. Molecular docking analysis demonstrated that the binding free energies of IBC and KRE6, SKN1, ERG11, AFR1, MCA1, DNM1, HOB1, and GAT201 were − 9.7 kcal/mol, − 7.0 kcal/mol, − 8.4 kcal/mol, − 7.5 kcal/mol, − 7.4 kcal/mol, − 7.9 kcal/mol, − 6.8 kcal/mol, and − 7.0 kcal/mol, respectively, and these interactions exhibited stronger binding affinity. Among these interactions, the docking score of IBC and ERG11 was the lowest, indicating the strongest binding affinity. Molecular docking experiments reveal that IBC strongly targets the protein Erg11, suggesting that the antifungal mechanism of IBC may be similar to azoles, which are fungistatic against *Candida* and work by inhibiting Erg11.

**Fig. 8:**
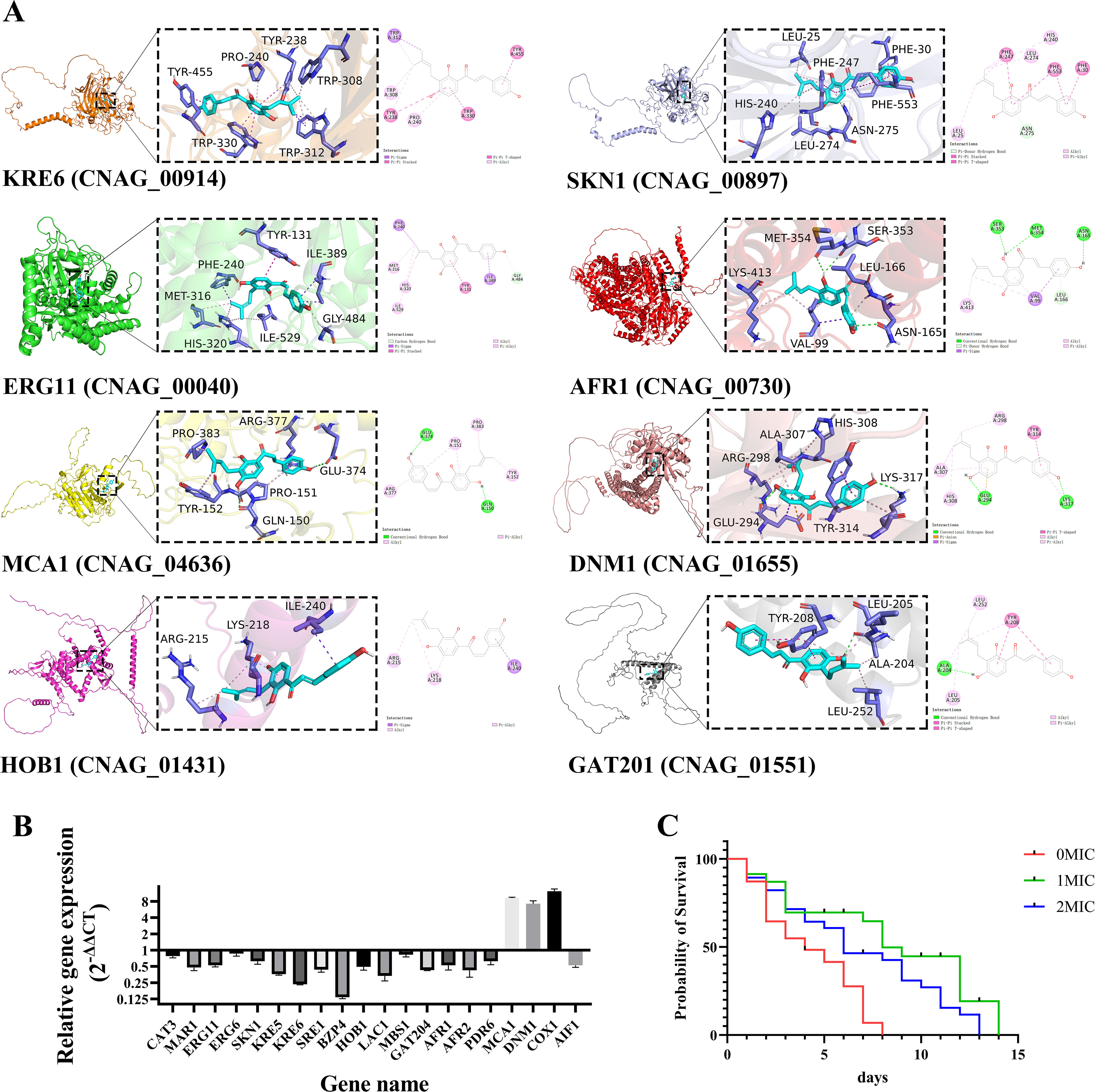
(**A**) Detailed diagram of molecular docking between IBC and target proteins, including KRE6, SKN1, ERG11, AFR1, MCA1, DNM1, HOB1 and GAT201. The 2D and 3D intermolecular contact between IBC and target proteins. Chemical structures were drawn by ChemDraw Pro 16.0 Suite (PerkinElmer, USA) and analyzed by the Discovery studio visualizer. IBC was represented in light blue, and local magnification of the docking sites and 2D diagram of the interactions were shown. (**B**) RT-qPCR assay for representative DEGs to validate the result of functional enrichment analysis. Data in the bar graphs are presented as the means and standard deviations of three independent experiments. (**C**) Effect of IBC on the lifespan of *C. neoformans*-infected *C. elegans* nematodes was evaluated, and the survival curve was plotted. Survival of *C. elegans* was evaluated in different treatment groups. Values represented the mean survival rates of three biological replicates

Moreover, Fig. 8A further showed the binding conformation between IBC and target proteins. Among these, IBC interacted with two amino acids labeled in purple in the SKN1 model, resulting in hydrogen bond interactions with SEP-54 and ASP-53, whose hydrogen bond distances were 2.2 and 2.4, respectively. IBC interacted with three amino acids labeled in purple in the ERG11 model, which had hydrophobic interactions with ALA-435, hydrogen bond interactions with VAL-439, and π bond interactions with LYS-440. IBC interacted with three amino acids labeled in purple in the MCA1 model, which formed hydrogen bond interactions with GLN-301, LYS-208, and MET-197, whose hydrogen bond distances were 2.4, 2.7, and 2.4, respectively. IBC interacted with five amino acids labeled in purple in the HOB1 model, which formed hydrophobic interactions with GLU-23, LIE-20, PHE-186, and ARG-190 as well as hydrogen bond interactions with ARG-189, and whose hydrogen bond distances were 2.1 and 2.6, respectively.

### Validation of RNA-Seq data by RT-qPCR analysis

In assessing the expression levels of target genes, RT-qPCR is considered the most accurate and commonly used method. The accuracy of RNA-Seq expression data was validated by comparing aliquots of the same total RNA samples used for RNA-Seq through RT-qPCR analysis. They were randomly selected for RT-qPCR examination, including cell wall/membrane biosynthesis-related genes (Kre5, Kre6, Skn1, Erg6, and Erg11), drug resistance-related genes (Afr1, and Afr2), virulence-related genes (Lac1, Hob1, and Bzp4), and mitochondrial genes (Dnm1, and Aif1). The DEGs from RNA-Seq were compared using RT-qPCR analysis, and the results of RT-qPCR were strongly correlated with those of the RNA-Seq, demonstrated the RNA-Seq data reliability (Fig. 8B). In order to validate the results of phenotypic assays and to explore the mode of antifungal and antibiofilm activity of IBC at the molecular level, RNA-seq and RT-qPCR analysis were performed for cell wall/membrane, drug resistance, and mitochondria-related genes associated with cell death of *C. neoformans* H99. The fungal cell wall of *C. neoformans* H99, composed mainly of chitin, glucans, and glycoproteins, is an essential component for growth and viability and absent from mammalian cells. Among them, the polysaccharide β-1,6-glucan is the most component abundant β-glucan in the *C. neoformans* H99 cell wall [40]. Previous study demonstrated the key role of Kre5, Kre6 of the KRE family and Skn1 on the β-1,6-glucan synthesis, maintenance of cell wall integrity and retention of mannoproteins and known cryptococcal virulence factors in the *C. neoformans* H99 cell wall. The Kre5 deletion alone or in combination with Kre6 and Skn1 resulted in the loss of cell wall β-1,6-glucan, accompanied by disruption of growth, morphology, cell wall integrity and virulence [59].

Meanwhile, RT-qPCR analysis of Kre5 and Kre6 genes further validated the results of RNA-seq. Similarly, the improved susceptibility of IBC-treated *C. neoformans* H99 to cell wall stressors could be attributed to the down-regulation of the Kre5, kre6 and Skn1 genes. In addition, previous study showed that disruption of Kre5 or Kre6 and Skn1 in combination affected the size and architecture of the exopolysaccharide capsule, a primary *C. neoformans* H99 virulence factor, and consistent with the results of capsule quantification assay [71]. Consequently, the key enzymes involved in the biosynthesis of β-1,6-glucan in the fungal cell wall might be explored for the development of antifungal agents, including glucan synthase, chitin synthase. Previous study showed that the deletion of Hob1 or Sre1 substantially reduced resistance to cell wall/membrane stresses [72]. Here, IBC downregulated the expression of *C. neoformans* H99, and IBC treatment potentiated the susceptibility of *C. neoformans* H99 to cell wall stresses. Interestingly, the results of RNA-seq and RT-qPCR analysis showed the down-regulation of 13 genes related to the ergosterol biosynthesis (Erg3, Erg6 and Erg11) in the presence of 1 MIC IBC, and subsequently inhibited remarkably the ergosterol production of *C. neoformans* H99, as evidenced by the results of ergosterol quantification assay. Consistently, previous studies demonstrated that deficiencies in ergosterol biosynthesis resulted in impaired cell membrane integrity, cell polarization, cell fusion and cell wall assembly, and it was consistent with the results of cell viability assay [73]. In addition, the single inactivation of Erg genes in the late ergosterol biosynthesis pathway is lethal under normal growth conditions of ergosterol deprivation. Similarly, Hob1 deletion, a key regulator of ergosterol gene expression, decreased the induction of Erg2, Erg3, Erg5, Erg11 and Erg25 genes under sterol depletion [74]. These results further indicate that IBC acts on several key genes related to the biosynthesis of ergosterol, exerting its antifungal effect by impacting the synthesis of major membrane ergosterol and thereby influencing membrane permeability.

IBC upregulated the expression of Dnm1, Fis1 and Mdv1 genes, and downregulated the expression of Aif1 gene. According to a previous report, the mutant cells with single deletion of Dnm1, Fis1 and Mdv1 were defective in fission, manifested as extremely high levels of tubular morphology relative to the wild type strain [31]. By contrast, the up-regulation of the Dnm1, Fis1 and Mdv1 genes by IBC could trigger the improved levels of diffuse morphology of *C. neoformans* H99 in the presence of IBC. In addition, deletion of Aif1 causes attenuated apoptosis in *C. albicans* under apoptosis-inducing conditions [65]. Previous study showed that in *Saccharomyces cerevisiae*, a hyperosmotic stress stimulus could trigger an apoptosis-like programmed cell death that is mediated by a caspase-dependent mitochondrial pathway partially dependent on cytochrome c [75]. Here, the results of RNA-seq and RT-qPCR analysis demonstrated the down-regulation of the Aif1 gene and the up-regulation of cytochrome c gene, which was consistent with results of the apoptosis assay. These results suggested that IBC plays an antifungal role by mediating mitochondria damage, leading to fungal cell death. In addition, the results of RNA-seq and RT-qPCR analysis demonstrated the down-regulation of the Afr1, Afr2, and Afr3 genes and the up-regulation of cytochrome c gene, which was consistent with results of the combinatorial drug assay [76]. The combinatorial drug therapy has an advantage of decreasing the antifungal drug dosage, thereby reducing drug toxicity and development of antifungal resistance.

### IBC enhanced the *C. Neoformans*-infected *C. Elegans* lifespan

The in vivo antifungal activity of IBC was assessed by examining its effect on the lifespan of *C. neoformans*-infected N_2_ worms. Figure 8C shows that the mean lifespan of *C. neoformans-*infected N_2_ worms was 7.6 ± 0.1 days without IBC. However, when treated with 1 MIC and 2 MIC IBC, *C. neoformans*-infected N_2_ worms lived for an average of 14.3 ± 0.5 days and 13.4 ± 0.2 days, respectively. A significant extension of the mean lifespan by 88.15% and 76.31% compared to that of the control group was obtained, respectively. These results observed that the increased lifespan of *C. neoformans*-infected *C. elegans* was proportional to a dose-dependent manner, which could be attributed to the potent in vivo antifungal effects exerted by IBC. Furthermore, a previous study has demonstrated that 25 µg/mL IBC was not able to reduce the cell viability of HaCaT cells after 24 h, implying that IBC exhibits low cytotoxicity and is safe for host cells [77].

### IBC-induced cell death mechanism in *C. Neoformans* H99 cells

**Fig. 9:**
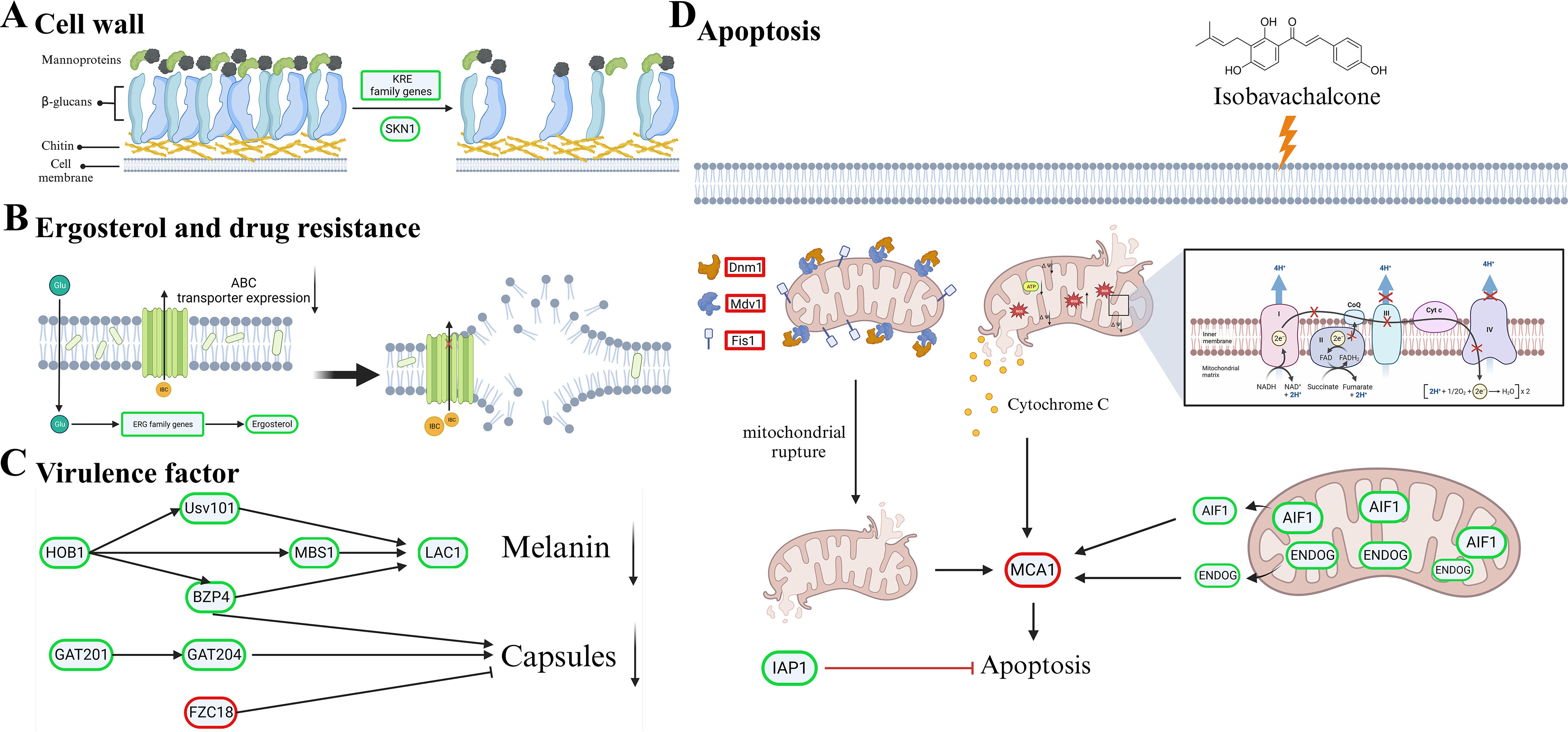
The mechanism diagram of IBC-induced cell death mechanism in *C. neoformans* cells. (**A**) IBC exerted antifungal effects through impeding the cell wall biosynthesis by downregulating expression of most of key genes within the KRE family such as Kre5, Kre6, and Skn1 gene. (**B**) IBC treatment caused damage to cell membrane-mediated *C. neoformans* cell death by downregulating the expression of most of ergosterol-related (ERG) genes including Erg3, Erg6, and Erg11, and the ABC transporter gene such as Afr1 and Afr2, thereby disrupting the cell membrane integrity and reversing drug resistance. (**C**) IBC exposure decreased the melanin and capsule synthesis by downregulating Hob1, Usv101, and Lac1 and Gat201. (**D**) IBC treatment disrupted mitochondrial homeostasis by upregulated the expression of Dnm1, Mdv1 and Fis1 and Mca1 genes, and downregulated the expression of Aif1, which resulted in the mitochondrial-mediated apoptosis of *C. neoformans*. Genes circled in red and green are significantly upregulated and downregulated, respectively

IBC demonstrated antifungal and antibiofilm effects against *C. neoformans* H99 by activating various regulated cell death pathways. These included damaging cell wall/membrane integrity, disrupting mitochondrial homeostasis, and reducing the production of ABC transporter and virulence factor. Figure 9A shows a significant downregulation in expression of Kre5, Kre6 and Skn1 genes, which was essential for the synthesis β-1,6-glucan. The decrease in β-1,6-glucan in kre6/skn1 or kre5 mutants adversely affected the growth, morphology and cell integrity, which was validated by FESEM and TEM analysis as well as cell phenotype experiment results. Simultaneously, the expression of 13 ERG genes, including key Erg3, Erg6, and Erg11, was significantly downregulated in response to IBC, inhibiting the ergosterol production, thereby leading to the damage to cell membrane integrity, which was consistent with SYTO 9/PI double staining assay (Fig. 9B). The expression of ABC transporter such as Afr1, and Afr2 was also downregulated, restoring the effectiveness of existing drugs (Fig. 9B). In addition, IBC downregulated the expression of melanin-related genes (Hob1, Usv01, Mbs1, Bzp4 and Lac1) and Gat201, and upregulated Fzc18, thereby decreasing the production melanin and capsules (Fig. 9C). Significantly, mitochondrial homeostasis, including the alterations of mitochondrial morphology, the MMP and ATP, ROS production, and oxidative phosphorylation were disrupted in IBC-treated *C. neoformans* H99 cells. The changes in apoptosis-related Aif1 and Mca1 genes caused apoptosis, induced cell death of *C. neoformans* H99 (Fig. 9D). The mechanisms of the antifungal and antibiofilm compound IBC against *C. neoformans* H99 include the damage to the integrity of the fungal wall/membrane, mitochondrial homeostasis and the induction of apoptosis, thereby resulting in the death of *C. neoformans* H99.

## Conclusion

This study exhibited the antifungal and antibiofilm efficacy of IBC against *C. neoformans* H99. The results of phenotypic assays and RNA-seq analysis provide important insights into the fungicidal effectiveness of IBC employing multiple antifungal mechanisms for *C. neoformans* H99. Nevertheless, future molecular characterization of key genes in response to IBC is required to exploit promising new antifungal agents with clear targets. In current work, the necessity of novel antifungal agents for effective treatment of *C. neoformans* H99 infections caused by *C. neoformans* H99 pathogen is underscored. The investigation revealed that IBC exhibited a MIC of 8 µg/mL against *C. neoformans* H99, and effectively disrupted 48-h mature biofilms at a concentration of 16 µg/mL by influencing cell viability. The antifungal potential of IBC was further confirmed through microscopic evaluations employing specific dyes and in vitro assays, which demonstrated the disruption of cell wall and membrane integrity, primarily by inhibiting the synthesis of ergosterol and β-1,6-glucan. RNA-Seq analysis was conducted to elucidate the influence of IBC on the cellular transcriptome of *C. neoformans* H99. The significant changes induced by IBC were evident in the RNA sequencing data. The transcriptomic findings were further investigated using RT-qPCR, which assisted in identifying DEGs. The results indicated that IBC impeded the growth, biofilm formation, and virulence of *C. neoformans* H99 by modulating multiple dysregulated pathways related to cell wall and membrane integrity, drug resistance, apoptosis, and mitochondrial homeostasis. These transcriptomic insights were supported by additional analyses, including antioxidant assays, antifungal drug sensitivity testing, molecular docking, and assessments of capsule and melanin production. Furthermore, the in vivo antifungal efficacy of IBC was demonstrated through experimentation on *C. elegans*, where IBC treatment resulted in a remarkable increase in the lifespan of *C. neoformans*-infected nematodes. In summary, this comprehensive study revealed that IBC targeted multiple pathways simultaneously, significantly inhibiting growth, biofilm formation, and virulence, in addition to dispersing mature biofilms and inducing cell death in *C. neoformans* H99.

### Electronic supplementary material

Below is the link to the electronic supplementary material.

Supplementary Material 1

### Electronic supplementary material

Below is the link to the electronic supplementary material.

Supplementary Material 2

## Data Availability

No datasets were generated or analysed during the current study.
