# Supplementary material for: Deciphering antifungal and antibiofilm mechanisms of isobavachalcone against Cryptococcus neoformans through RNA-seq and functional analyses
Source: Microb Cell Fact. 2024 Apr 12;23:107. doi: 10.1186/s12934-024-02369-2 (PMC11015616; doi:10.1186/s12934-024-02369-2)
Supplement: Supplementary file 1 — Supplementary Material 1 [file 12934_2024_2369_MOESM1_ESM.doc]

Supplementary material

**Fig. S1** Killing-growth curve analysis of *C. neoformans* cells in the absence and presence of 1MIC IBC. *C. neoformans* (5 replicates) was grown in a deep 24-well plate at 30 °C. For determining the growth, 200 μL were aspirated every 2 h and monitored as OD_600_ for 24 h using the growth reader under constant shaking. The growth curve was prepared based on the growth of three independent cultures, and the mean ± SD was plotted.

**Fig. S2** (A) Statistical map of gene expression value distribution of each sample. The abscissa is the sample name (I-H99, the IBC-treated group), and the ordinate is log10 (FPKM). (B) The volcano map analysis of DEGs expression level in untreated versus IBC-treated *C. neoformans* cells. The blue, gray and red nodes represented downregluation, not different and upregluation, respectively.

**Fig. S3** Protein interactions were analyzed using the STRING database and networkX in Python, resulting in interactions networks on ergosterol, virulence factors, drug resistance mechanisms, and mitochondrial apoptosis.

**Fig. S4** The combination of fluconazole (FLC) and IBC displays synergistic effect against *C. neoformans.*
