# Supplementary material for: Deciphering antifungal and antibiofilm mechanisms of isobavachalcone against Cryptococcus neoformans through RNA-seq and functional analyses
Source: Microb Cell Fact. 2024 Apr 12;23:107. doi: 10.1186/s12934-024-02369-2 (PMC11015616; doi:10.1186/s12934-024-02369-2)

Table S1: Primers sequences used for qRT-PCR in this study

| Primer | Sequence 5’-3’ | Reference |
| --- | --- | --- |
| CAT3 | CTGATTGTGAGGCTCGGGTT | This study |
|  | TGATACAAGTACAGCCGCCAA |  |
| MAR1 | ATGTCCAACCGTCACTGTCC |  |
|  | CTTACCATGGGTTGGCTCGT |  |
| ERG11 | CAGCCATGTCGGCAATCATC |  |
|  | CCTTCCCCTGGAGAGTACGA |  |
| ERG6 | CAATAACAATGCCCGCCGAA |  |
|  | AGTGGCATCAGAGAAACGGG |  |
| SKN1 | GGTACGGTGCCTCAACAGAA |  |
|  | ATACCCCTGATCCCTTGCCT |  |
| KRE5 | CATTTTCGCCCTGGACCTCT |  |
|  | AATGTCTTCGGGGATTGCGT |  |
| KRE6 | AAGGCGACTCATTCCCCAAG |  |
|  | CATCGGGGTCACATCCAACA |  |
| SRE1 | GGATCTTTACGGGGCCTAGC |  |
|  | CTCAGATTCCACACCCACCC |  |
| BZP4 | CCAGCTCGTCTTTCCCAAGT |  |
|  | CCACCTCTCCATTTGGCCTT |  |
| HOB1 | TGGAGGGCAAGAACGAAGAC |  |
|  | AACTTGCGAGGTAACTGGGG |  |
| LAC1 | CCGATACCCGATAGCTCCCT |  |
|  | CCCCGCATTACTGTGAGTGT |  |
| MBS1 | TCAAGAACTTGCCGACGTGA |  |
|  | CGGTGGGAACATCCCTCAAA |  |
| GAT204 | GAGCCGGCGATCTTTTGTTC |  |
|  | GCATGTGAGTGGTTGTGCTG |  |
| AFR1 | CGATTTGCCCTTGCCACAAA |  |
|  | CGACTCCCTTGTGCTTCTGT |  |
| AFR2 | TTGGACCACGTGGATGGATG |  |
|  | GTTGTTGCCAAACCCTCGTC |  |
| PDR6 | GTTGTTGCCAAACCCTCGTC |  |
|  | GCTGTCCACCTACTTTGCCT |  |
| MCA1 | TATGGCTACAGACCCCCTCC |  |
|  | CTGGTGGCTGGTATTGCTGA |  |
| DNM1 | CATTCACACACCTCCTCGCT |  |
|  | AGTTCCCTCCCCCATAGACC |  |
| COX1 | TGGCTTTTCTCAACCAACGC |  |
|  | CCATCCCGAGGTAGCCAAAA |  |
| AIF1 | GAACCAGCCATCCCTAACCC |  |
|  | GAACGGAGACGCGATTGTTG |  |


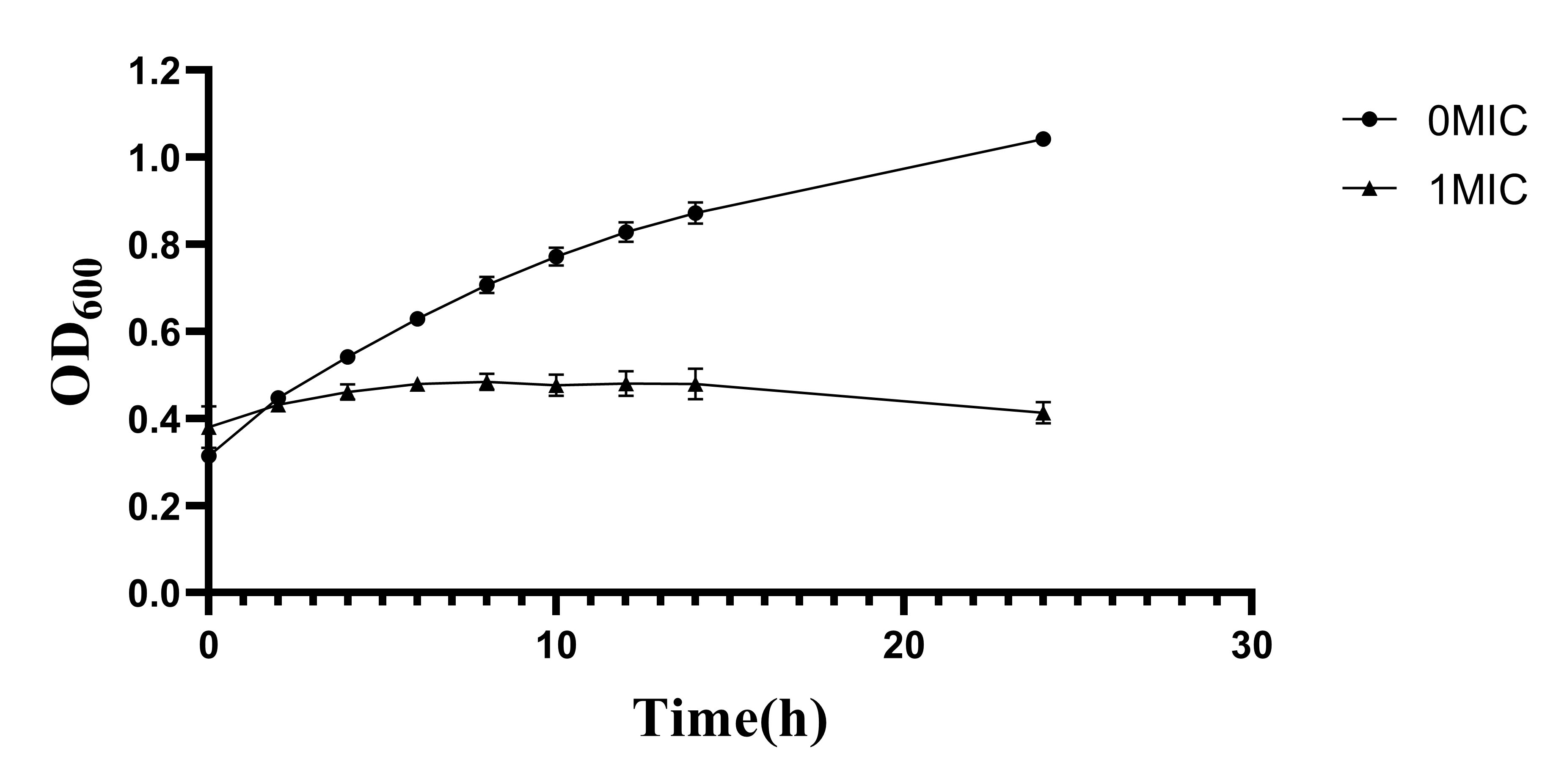


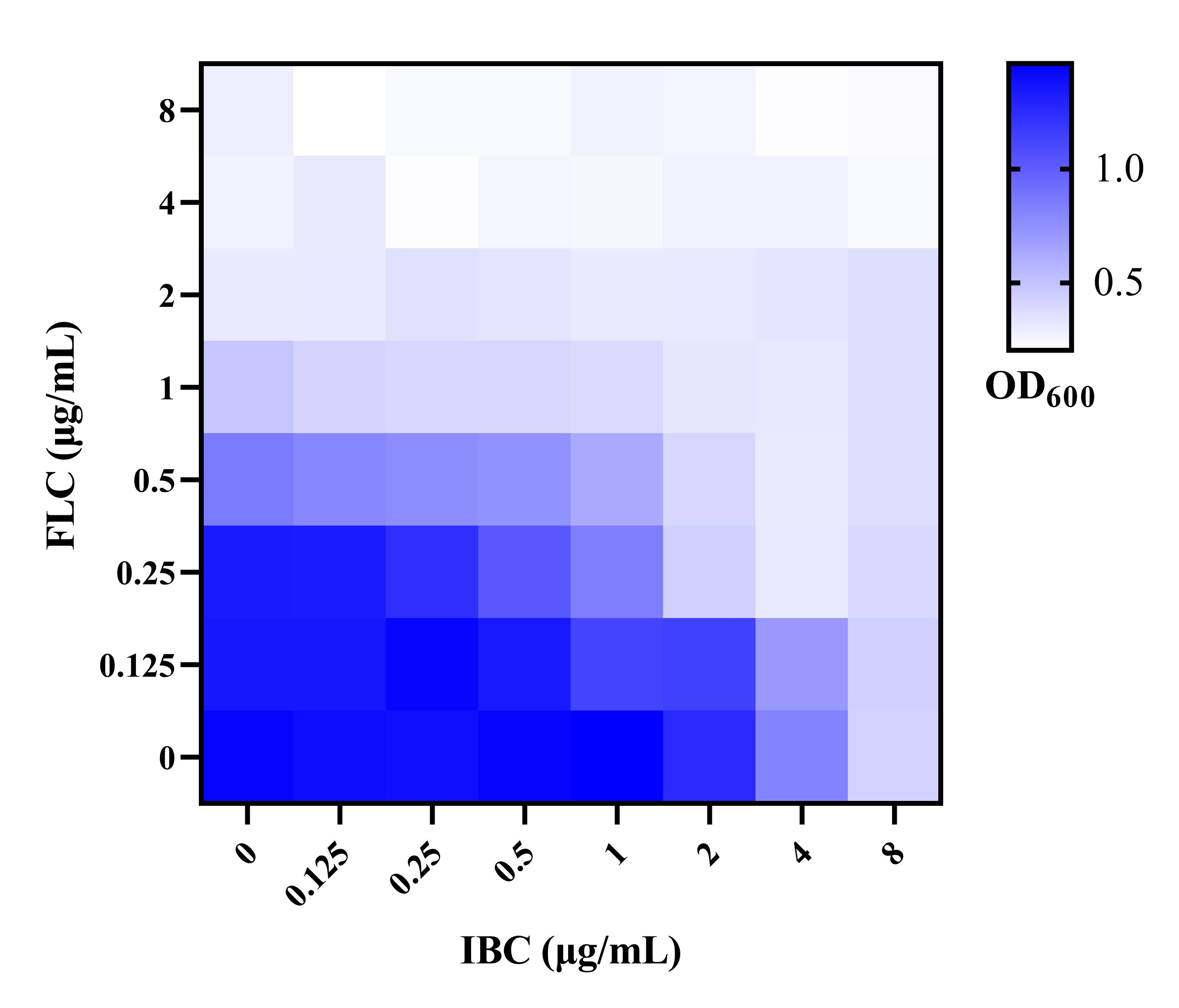

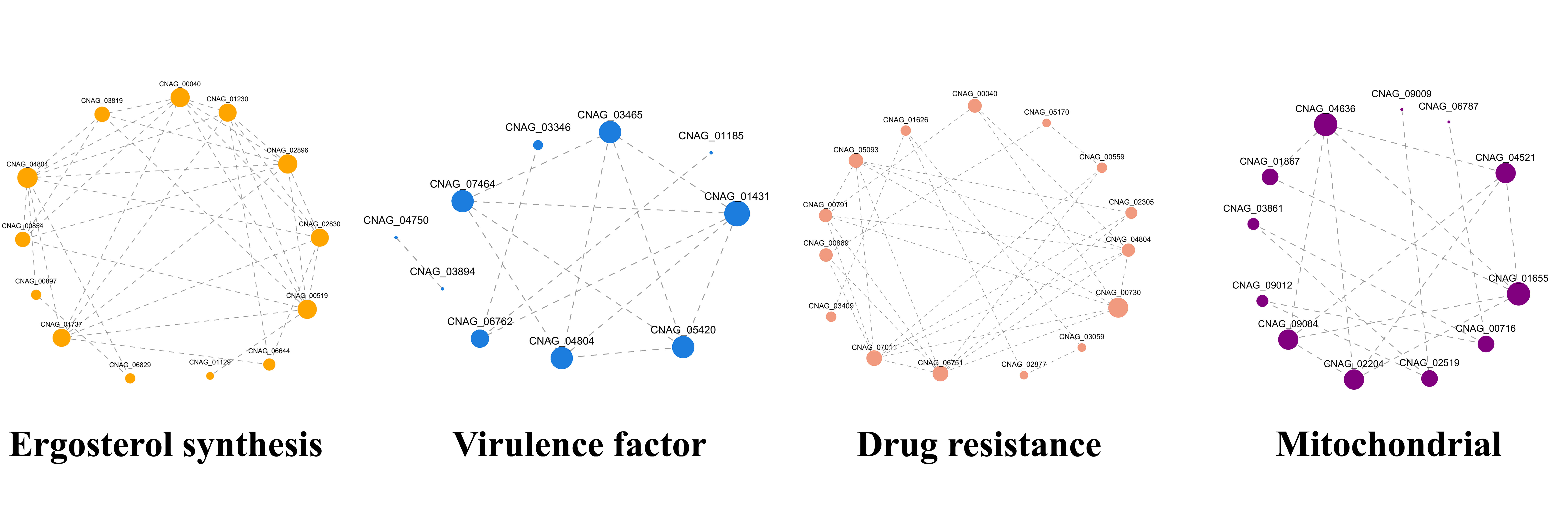


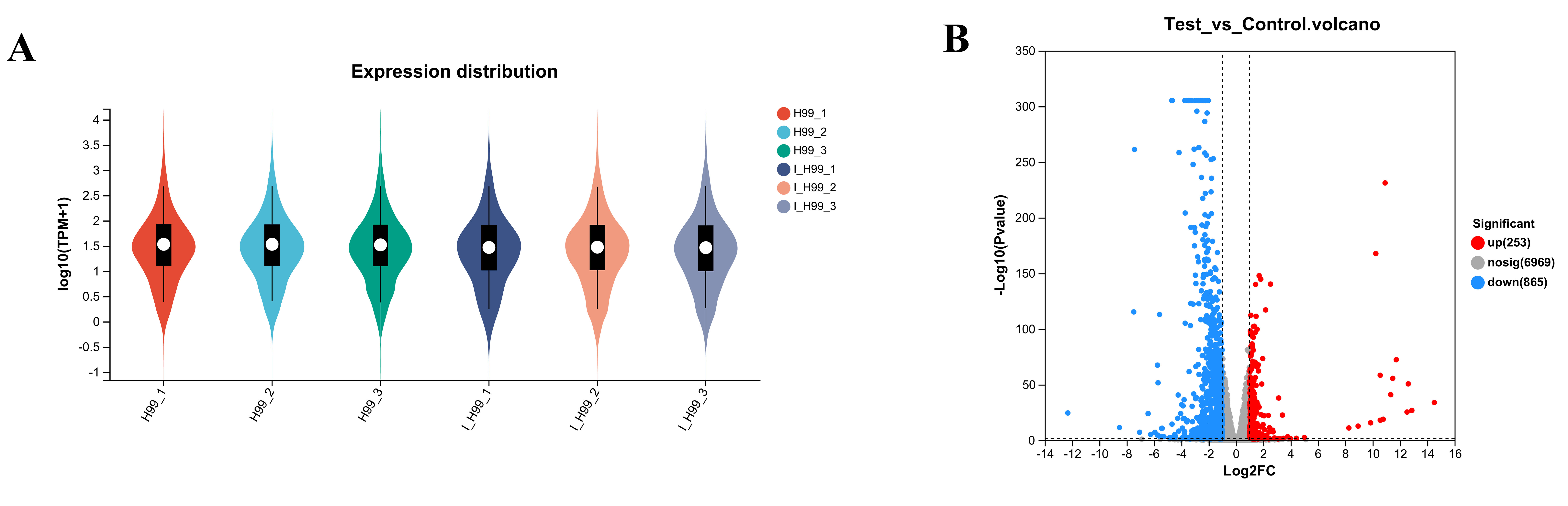

Supplement: Supplementary file 2 — Supplementary Material 2 [file 12934_2024_2369_MOESM2_ESM.doc]
